# 2014 AAPM Spring Clinical Meeting ‐ Abstracts

**DOI:** 10.1120/jacmp.v15i3.4958

**Published:** 2014-05-08

**SATURDAY, MARCH 15**

**Best Poster Competition Exhibit Hall**

**PO‐BPC‐Exhibit Hall‐01**

## The Clinical Impact of Detector Choice for Beam Scanning

J Gersh,^1*^ R Best,^2^ R Watts^3^

*Gibbs Cancer Center & Research Institute ‐ Pelham,^1^ Greer, SC, University of Virginia Health Systems,^2^ Charlottesville, VA, International Medical Physics Services,^3^ San Antonio, TX*

### Purpose

Recently, the developers of Eclipse have recommended the use of ionization chambers for all profile scanning, including for the modeling of VMAT and stereotactic applications. The purpose of this study is to show the clinical impact caused by the choice of detector with respect to its ability to accurately measure dose in the penumbra and tail regions of a scanned profile.

### Methods

Using scan data acquired with several detectors including an IBA CC13, a PTW 60012, and a Sun Nuclear Edge detector, three complete beam models are created, one for each respective detector. Next, using each beam model, dose volumes are retrospectively recalculated from actual anonymous patient plans. These plans include three full‐arc VMAT prostate plans, three left chest wall plans delivered using irregular compensators, two half‐arc VMAT lung plans, three MLC‐collimated static‐field pairs, and one SBRT liver plan. Finally, plans are reweighted to deliver the same number of monitor units, and mean dose to target volumes and organs at risk are calculated and compared.

### Results

Penumbra width did not play a role. Dose in the tail region of the profile made the largest difference. By overresponding in the tail region of the profile, the 60012 diode detector scan data affected the beam model in such a way that target doses were reduced by as much as 0.4% (in comparison to CC13 and Edge data). This overresponse also resulted in an overestimation of dose to peripheral critical structure whose dose consisted mainly of scatter.

### Conclusion

This study shows that for modeling the 6 MV beam of Acuros XB in Eclipse Version 11, the choice to use a CC13 scanning ion chamber or an Edge detector was an unimportant choice, providing nearly identical models in the treatment planning system.

**PO‐BPC‐Exhibit Hall‐02**

## Surface Dose Effects of Linen Coverings for Breast and Chest Wall Patients

J Fagerstrom,^1*^ E Hirata^2^

*University of Wisconsin,^1^ Madison, WI, Queens Medical Center,^2^ Honolulu, HI*

### Purpose

To evaluate the effect of various linen patient coverings on surface dose of breast and chest wall patient geometry.

### Methods

TLDs were placed on the chest wall surface of an anthropomorphic male RANDO phantom that was irradiated with photon tangent beams and electron enface fields with a circular block. The measurements were repeated with the phantom covered with a hospital gown, sheet, towel, all three items together, and without any coverings. Photon and electron beams of varying energies were used to reproduce typical patient treatments.

### Results

There was an increase in measured surface dose due to the gown between 4.6%±2.1% and 6.9%±2.7% for photons, and 0.0%±3.6% to 3.0%±2.2% for electrons. The TLD readings showed an overall increase in dose with increasing thickness of covering for photons; however, electron results did not show a definitive relationship between surface dose and covering thickness due to statistical uncertainties associated with the TLD readings.

### Conclusion

While further studies are needed to quantify the effect, initial TLD results suggest that draping patients with linen coverings may increase skin dose. An increase in surface dose was clear with photon irradiations mimicking patient treatments, though similar electron irradiations demonstrated inconclusive results.

**PO‐BPC‐Exhibit Hall‐03**

## Using Failure Mode and Effects Analysis to Determine the Incident Learning System Clinical Action Scale

J Johnson,^*^ M Gillin, S Bilton, L Court, A Evans, S Harrelson, S Hayden, J Kanke, S Kirsner, M Palmer, B Riley, P Sackett, J Tonigan, G Walker, X Zhu, P Das, G Ibbott

UT MD Anderson Cancer Center, Houston, TX

### Purpose

Published incident learning consensus recommendations allow incidents to be classified at the local clinic level using an internally developed clinical action scale. A large, academic radiation oncology practice used failure mode and effects analysis (FMEA) as a multidisciplinary, systematic, and consensus approach to revise its local clinical action scale.

### Methods

From September to December 2013, a multidisciplinary team was formed, consisting of medical physicists, dosimetrists, therapists, and radiation oncologists. The team met as a group, and individually completed an FMEA designed survey. The survey consisted of 81 published incident learning structure process steps, each with a 1–10 scale for the likelihood of occurrence (O), detectability (D), and severity (S). Completed surveys were used to calculate the average risk priority number (RPN) (O×D×S) for each process step; the process steps were ranked from highest to lowest RPN and evenly divided into three relative risk groups (high, intermediate, and low). Through discussion, the team made minor adjustments and finalized its local clinical action scale.

### Results

The time required for each team member was approximately 2 and 5 hours for surveys and discussion, respectively. The process step average RPNs ranged from 194.3 (highest) to 28.3 (lowest). 81 process steps were evenly divided into three relative risk groups: high (RPNs 194.3 to 93.5), intermediate (RPNs 91.4 to 74.3), or low (RPNs 74.1 to 28.3). Review and discussion of the groupings showed high agreement, with only ten recommended group changes, corresponding to 12% of the process steps.

### Conclusion

FMEA is an effective way for a multidisciplinary team to systematically come to a consensus and determine the local clinical action scale in the incident learning system. Although there is variability in the actual RPN value given to individual processes, there is much more agreement on whether each process is high, intermediate, or low relative risk.

**PO‐BPC‐Exhibit Hall‐04**

## Quantifying Isocenter Measurements to Establish Clinically Meaningful Thresholds

T Denton,^1,2*^ L Shields,^3,4^ J Howe,^1,2^ A Spalding^3,4,5^

*The Norton Cancer Institute Radiation Center,^1^ Louisville, KY, Associates In Medical Physics, LLC,^2^ Greenbelt, MD, Norton Neuroscience Institute,^3^ Louisville, KY The Brain Tumor Center,^4^ Norton Healthcare, Louisville, KY, Kosair Children 's Hospital,^5^ Louisville, KY*

### Purpose

A range of isocenter congruency verification tests have been examined from a statistical perspective for the purpose of establishing tolerance levels that are meaningful, based on the fundamental limitation of linear accelerator isocentricity and the demands of a high‐precision stereotactic radiosurgery program.

### Methods

Using a laser‐defined isocenter, 149 individual isocenter congruency tests were examined with recorded values for ideal spatial corrections to the isocenter test tool. These spatial corrections were determined from radiation exposures recorded on an electronic portal imaging device (EPID) at various gantry, collimator, and treatment couch combinations. The limitations of establishing an ideal isocenter were quantified from each variable which contributed to uncertainty in isocenter definition. Individual contributors to uncertainty, specifically, daily positioning setup errors, gantry sag, multileaf collimator (MLC) offset, and couch walkout, were isolated from isocenter congruency measurements to determine a meaningful isocenter measurement.

### Results

Variations in positioning of the test tool constituted, on average, 0.38 mm magnitude of correction. Gantry sag and MLC offset contributed 0.4 and 0.16 mm, respectively. Couch walkout had an average degrading effect to isocenter of 0.72 mm. Considering the magnitude of uncertainty contributed by each uncertainty variable and the nature of their combination, an appropriate schedule and immediate action level were determined for use in analyzing daily isocenter congruency test results in a stereotactic radiosurgery (SRS) program.

### Conclusion

The recommendations of this study for this linear accelerator include a schedule action level of 1.25 mm and an immediate action level of 1.50 mm requiring prompt correction response from medical physicists before SRS or stereotactic body radiosurgery (SBRT) is administered. These absolute values were derived from considering relative data from a specific linear accelerator and, therefore, represent a means by which a numerical quantity can be used as a test threshold with relative specificity to a particular linear accelerator.

**PO‐BPC‐Exhibit Hall‐05**

## Experimental Validation of a Correction‐Less Diode for Small Field Reference Dosimetry

P Charles,^1^ G Cranmer‐Sargison,^2*^ J Trapp,^1^ D Thwaites^3^

*Queensland University of Technology,^1^ Brisbane, Saskatoon Cancer Centre,^2^ Saskatoon, SK, University of Sydney,^3^ Camperdown, Sydney*

### Purpose

A recent Monte Carlo based study has shown that it is possible to design a diode detector that produces measured relative output factors that are equivalent to that in water.^(1)^ The aim of this work was to test this concept experimentally, using two different unshielded diodes.

### Methods

The diode detectors selected were the PTW T60017 electron diode and a custom made Sun Nuclear unshielded diode. A plastic, water‐tight, cap was constructed so that it could be placed over the proximal end of the diode to provide varied air gap thicknesses. All measurements were made on Varian iX accelerators at a nominal 6 MV beam energy for square fields of side 0.5 cm to 5.0 cm. The PTW electron diode measurements were made with the air gap thickness set to 0.0, 0.5, 1.0, and 1.5 mm. The Sun Nuclear unshielded diode measurements were made with the air gap thickness set to 0.0, 0.3, 0.6, and 0.9 mm. Stereotactic field diode measurements corrected using published Monte Carlo calculated factors were used as a benchmark.

### Results

The optimal air thickness required for the PTW T60017 electron diode was 1.0 mm. This was close to the Monte Carlo predicted value of 1.15 mm. The sensitivity of the “correction‐less” PTW T60017 diode design was independent of field size to within ±1%. The Sun‐Nuclear unshielded diode results were more susceptible to small variations in the practical air gap thickness, however did provide field size independence within the experimental uncertainty.

### Conclusion

Existing commercial diode detectors can be converted into a “correction‐less” small field dosimeter using the simple addition of an air cap. The method of applying a cap to create a small field diode does provide a dual purpose detector as without the cap, the diode is still an unmodified electron diode.

1. Charles et al. Monte Carlo‐based diode design for correction‐less small field dosimetry. Phys Med Biol. 2013;58:4501‐12.

**PO‐BPC‐Exhibit Hall‐06**

## How Do Reconstruction Algorithms and Radiation Doses Affect the Accuracy of CT HU?

C Dodge,^*^ J Rong

*MD Anderson Cancer Center, Houston, TX*

### Purpose

To determine the potential change in Hounsfield units (HU) with adaptive statistical iterative reconstruction (ASiR) and model‐based iterative reconstruction (MBiR), as compared to conventional filtered back‐projection (FBP) reconstruction methods.

### Methods

The sensitometry CTP404 module of a Catphan 600 phantom was scanned using a GE Discovery HD750, at 120 kVp, 0.8 sec rotation time, and pitch factors of 0.516, 0.984, and 1.375. The mA was selected to achieve CTDIvol values of 24, 18, 12, 6, 3, 2, and 1 mGy. The module was inserted into a fat‐equivalent oval ring to better represent patient shape and size. Images were reconstructed in 2.5 mm thickness with FBP; 20%, 40%, and 70% ASiR; and MBiR. Eight cylindrical inserts in the module of the Catphan were used to quantify differences in HU: air, PMP, LDPE, background material, polystyrene, acrylic, Delrin, and Teflon. Slight variations from the estimated CT numbers in phantom manual were expected to result from use of the fat ring during scan acquisition.

### Results

MBiR‐reconstructed HU for the background (100 HU), PMP (‐200 HU), LDPE (‐100 HU), Polystyrene (‐35 HU), Acrylic (120 HU), Delrin (340 HU), Teflon (990 HU) and air (‐1000 HU) were closest in value to the expected HU outlined in the Catphan 600 manual, compared to FBP and ASiR. Across the three pitch factors, MBiR maintains its accuracy better than FBP and ASiR. HU accuracy for all materials and pitch factors dramatically decreases below 5 mGy CTDIvol. Conclusion: MBiR algorithms provide the most accurate estimation of HU compared to FBP and ASiR at dose levels higher than 5 mGy and across all pitch factors. The results provide guidance to clinical practice, especially in treatment planning for radiation therapy.

**PO‐BPC‐Exhibit Hall‐07**

## Measuring Output Factors for Photon Fields Smaller than 10 cm×10 cm

B Doozan,^1*^ S Pella,^2^ A Bacala,^1,3^ T Leventouri,^1^ C Smith^1^

*Florida Atlantic University,^1^ Boca Raton, FL, South Florida Radiation Oncology,^2^ Boca Raton, FL, Department of Physics,^3^ Mindanao State University‐Iligan Institute of Technology, Iligan City, Philippines*

### Purpose

To accurately measure the field output factors (OF) for photon beams from a 6 MV medical accelerator for small photon fields up to 10 cm×10 cm.

### Methods

Two diode detectors and three ionization chambers are each separately placed inside a scanning water phantom at two different positions: source‐to‐surface‐distance (SSD) of 100 cm at a depth of 10 cm and at SSD of 95 cm at 5 cm depth. The photon field is collimated either by the jaws or by the multileaf collimator (MLC). Regression analysis and analysis of variance are applied to the OF data versus detector volume for the 1 cm×1 cm field size.

### Results

For square fields of width greater than 3 cm, OF measurements from the detectors agree to within 1% or less. The largest variation in OF occurs for the 1 cm×1 cm field size, observed at any depth, SDD, and field collimation. For jaws‐shaped field, a difference of more than 18% is observed between the diode detector and the ionization chamber of the biggest volume. Additionally, there is a positive correlation between OF and detector sensitive volume where the regression coefficient is greater than 0.75 for all cases.

### Conclusion

Measured OFs are found to depend upon the field size, SSD, depth, and also upon the type of beam collimation, size, and type of detector used. The OF variation is maximum at small field sizes and when the detector volumes are orders of magnitude different. For a 5% significance level, where at least five detectors of different types are used in the OF measurements, the correlation between the OF and detector sensitive volume can be used to arrive at a corrected OF value at 1 cm×1 cm field size.

**PO‐BPC‐Exhibit Hall‐08**

## Superficial Photon and Electron Dose Measurements in Varying Bolus Materials

L Young^*^

*UW Medicine, Seattle, WA*

### Purpose

Bolus materials are commonly used to correct the depth of maximum (dmax) radiation dose deposition to the patient's skin surface. Brass mesh has been used as a substitute for Superflab or wet washclothes because it conforms to skin contours readily. A comparison study was conducted to determine how many layers or brass mesh would equate to 0.5 cm of water‐equivalent bolus for low‐energy photons and electrons.

### Methods

Superficial doses (0‐5 mm depth) for Superflab, wet washcloths, and varying layers of brass bolus were measured with a parallel plate ion chamber in a Solid Water phantom. With the surface of the phantom set to 100 cm SAD, electrometer readings of the bolus materials were compared and analyzed with 0.5 cm of solid water.

### Results

For 6 MV and 10 MV photons, three wash cloths and three layers of brass mesh produced skin doses that were within 2.4% and 5.1%, respectively, of solid water measurements. Superflab measurements were within 1.1% of solid water. In 6 MeV, 8 MeV, and 10 MeV electrons, one layer of brass mesh demonstrated a better match of skin doses in 0.5 cm solid water or Superflab. The percent difference in one‐layer and two‐layer brass mesh readings compared to solid water were −0.87% and 13.7%, respectively. The difference between Superflab and solid water was less than 1%.

### Conclusion

Three layers of brass bolus can be used as a suitable substitute for 0.5 cm of water‐equivalent bolus material in 6 MV and 10 MV photons. Although one layer of brass mesh is well matched with solid water at the surface, significant alterations in beam energy spectra increases the percent depth dose (PDD) profiles beyond dmax. Brass bolus should not be used with electrons without beam data revisions to account for changes in PDD profiles.

**PO‐BPC‐Exhibit Hall‐09**

## Automated, Real‐Time Evaluation of Radiation Treatment Plans for Protocol Constraints

RC Best,^1*^ JM Larner,^1^ K Wijesooriya,^1^ C Geesey,^1^ JA Gersh,^2^ B Libby^1^

*University of Virginia Health Systems,^1^ Charlottesville, VA, Gibbs Cancer Center & Research Institute ‐ Pelham,^2^ Greer, SC*

### Purpose

Automating evaluation of radiation therapy treatment plans for agreement with protocol constraints can reduce the time spent planning. Creating a treatment plan based on a protocol can be time‐consuming for dosimetrists due to the rigid dosimetric constraints which should be met. These values are typically gathered manually from the treatment planning system (TPS). If a dose constraint is not met, adjustment of the plan to meet one constraint requires rechecking of other constraints. This must be done in an iterative manner to meet all constraints.

### Methods

RTOG protocols 0915 and 0813 (peripheral and medial lung SBRT) require documentation of over 40 volumetric and dosimetric values. Scripts were developed in‐house that report contour volumes, create isodose contours, perform operations on contours, report region‐of‐interest (ROI) dose coverage, and report prescribed and maximum doses to ROIs. The results are reported immediately in a simple text document on the TPS computer. Included in the report are the current plan's values, the limit for each constraint, and a pass/fail indication for each.

### Results

Our current suite of scripts differs from existing solutions in both speed and capabilities. Third party DVH evaluation software requires export of contours and DICOM dose data, a cumbersome extra step. Our scripts have capabilities that go beyond current TPS limits, including creating isodose level contours, then performing Boolean operations on those contours. This is necessary for measuring the volume of the 105% isodose level outside the PTV. Additionally, our scripts can handle constraints which vary according to plan parameters. In both RTOG 0915 and 0813, the low dose spillage limits vary in steps according to tabular values of PTV volume.

### Conclusions

Our experience shows that automatically evaluating plans reduces planning time. Our scripts allow a faster and more complete evaluation than existing solutions.

**PO‐BPC‐Exhibit Hall‐10**

## Measurement of Interfractional Rotation of Pelvic Bony Anatomy: Assessment of Impact of BMI for Vac‐Lock System

L Song,^*^ C Mayo, I Petersen, M Haddock

*Mayo Clinic Rochester, Rochester, MN*

### Purpose

Develop an efficient approach to extend use of daily kV images already acquired to accurately and reproducibly measure daily variations in patient rotation during external beam treatment of genitourinary, gastrointestinal, and gynecologic disease, and use the approach to evaluate our current immobilization device (VacLock system) with respect to body mass index (BMI) as baseline for further patient immobilization optimization.

### Methods

We analyzed 214 orthogonal OBI kV image pairs acquired before daily radiation delivery for ten patients treated with 3D CRT, IMRT or RapidArc. Anatomic landmarks were identified that enabled reliable use of distances and angles measured between different landmarks on the kV images to calculate the yaw, pitch, and roll of the pelvis according to their geometric relationship. The correlation between rotational positioning errors and patient BMI and orientation (prone vs. supine) was investigated.

### Results

The patients in our sample covered a wide range of BMI (17.1−43.2). Mean values of pelvis rotation were 37.1%±8.5, 90.1%±1.4, 0.9%±1.5 for pitch, yaw and roll respectively. Comparing median standard deviation of the daily setup for each patient, all showed larger variation in the pitch angle $\left(1.4^{{}^{\circ}}\pm 0.6^{{}^{\circ}}\right)$ compared to the yaw (0.5±0.1) or roll (0.5±0.3). Data were suggestive that BMI had a larger impact in variation on roll than on pitch or yaw, but required more data at the extremes to quantify. Compared with supine patients, the two prone patients showed minimum yaw variation within their BMI group.

### Conclusion

KV image‐based anatomic metrics were useful as a tool to quantify daily variation in the patient rotation and defines a baseline for future comparisons with other immobilization systems. The range of motion of the six degree of freedom couches on our accelerators was adequate to compensate for the variations. Comparison between supine and prone patient needs further investigation.

**PO‐BPC‐Exhibit Hall‐11**

## Pulmonary Dosimetric Effects of a Moderate Deep Inspiration Breath‐Hold Technique for Left‐Sided Breast Cancer Patients

J Yu,^*^ D Brinkmann, S Park

*Mayo Clinic, Rochester, MN*

### Purpose

To evaluate the dosimetric impact of a moderate deep inspiration breath hold (mDIBH) on the ipsilateral lung for the whole breast (WB) treatment and the WB plus axillary nodes treatment, and to exam the applicability of a previously developed method of using an imaging‐based volumetric metric to indicate the received dose.

### Methods

This is a retrospective study including 25 patients. Among them, 17 patients received 3D CRT for left‐sided WB and eight patients were treated with WB plus axillary nodes. All patients except one had both mDIBH and FB CTs. Breast PTV‐eval was defined as per RTOG. For a given patient, two treatment plans were calculated retrospectively on FB and mDIBH CT images, and for the purpose of comparison, the same PTV coverage was achieved for both plans and the prescriptions were normalized to 50 Gy. Signed rank test was used for statistics evaluation.

### Results

Different from the published results on the WB treatments, in this study the left lung dose was found to be slightly increased with mDIBH, though the statistical differences have not been shown to be significant. The left lung dose was increased for half of the patients, while decreased for the other half. The preliminary data on the treatment of WB and axillary nodes showed that the increase of lung dose with mDIBH was even more obvious. Based on the previously developed method, linear correlations were also found between the exposed lung volumes and the lung dose, but the correlation was not strong. However, when the exposed volume was defined by the MLC, the correlation improved significantly.

### Conclusion

For the whole breast treatments with tangent FinF technique, mDIBH did not significantly affect the ipsilateral lung dose compared with FB. The geometrically defined exposed lung volumes by the MLC better represent the irradiated volumes in dosimetry than PTV‐based definition.

**PO‐BPC‐Exhibit Hall‐12**

## Interfraction Positional Variation in Pancreatic Tumors Using Daily Breath‐Hold Cone‐Beam Computed Tomography with Visual Feedback

M Nakamura,^1*^ M Akimoto,^1^ T Ono,^1^ S Yano,^2^ M Nakata,^2^ A Nakamura,^1^ S Itasaka,^1^ T Mizowaki,^1^ K Shibuya,^3^ M Hiraoka^1^

*Kyoto University Graduate School of Medicine,^1^ Kyoto, Kyoto University Hospital,^2^ Kyoto, Yamaguchi University Graduate School of Medicine,^3^ Yamaguchi, Japan*

### Purpose

To assess interfraction positional variation in pancreatic tumors using daily breath‐hold cone‐beam computed tomography at end‐exhalation with visual feedback (BH‐CBCT).

### Methods

Six consecutive patients with pancreatic cancer who underwent BH intensity‐modulated radiation therapy with visual feedback were enrolled. All participating patients stopped oral intake with the exception of drugs and water for >3 h before treatment planning and daily treatment. No patient was provided oxygen. Each patient was fixed in the supine position on an individualized vacuum pillow with both arms raised. The prescription dose was 45 to 51 Gy in 15 fractions. An isotropic margin of 5 mm was added to create the planning target volume. BH‐CBCT scans were performed before beam delivery in every fraction. BH‐CBCT acquisition was obtained in three or four times breath holds by interrupting the acquisition two or three times, depending on the patient's BH ability. The image acquisition time for a 360° gantry rotation was approximately 90 sec, including the interruption time due to BH. The initial setup errors were corrected based on bony structure, and the residual errors in the target position were then recorded. The magnitude of the interfraction variation in target position was assessed for 90 fractions.

### Results

The systematic and random errors were 1.5 and 2.1 mm, 1.2 and 1.8 mm, and 0.3 and 2.7 mm in the left‐right, anterior‐posterior, and superior‐inferior directions, respectively. Absolute interfraction variations of >5 and >10 mm were observed in eight (8.9%) and two (2.2%) fractions, respectively.

### Conclusion

BH‐CBCT with visual feedback provided high pancreatic tumor position reproducibility. However, absolute interfraction variations of >5 mm were occasionally observed. Therefore, target matching is required to correct interfraction variation, especially to ensure safe delivery of escalated doses to patients with pancreatic cancer.

**PO‐BPC‐Exhibit Hall‐13**

## Evaluation of Deterministic Grid‐Based Boltzmann Equation Solver for Dose Calculation in Endobronchial High‐Dose‐Rate Brachytherapy

M Axente,^1*^ S Wetherall,^2^ B Loo,^1^ A Sung,^1^ B Fahimian^1^

*Stanford Hospitals and Clinics,^1^ Stanford, CA, Varian,^2^ Crawley, West Sussex, UK*

### Purpose

To evaluate the use of deterministic grid‐based Boltzman (GBB) equation solvers for dose calculation in HDR endobronchial brachytherapy.

### Methods

Three lung cancer patients CTs and a virtual phantom composed of a $30\;{\text{cm}}^{2}$ block of lung with a 1 cm diameter central water column were used in the evaluation of dosimetric differences between TG‐43 formalism and BrachyVision (v.13) Acuros GBB solver. Materials were manually (phantom) or automatically assigned, based on the CT values (patients) from the Acuros library for inhomogeneous dose calculation. TG‐43 dose to water and Acuros dose to medium were calculated for both patient and phantom studies. The difference between the two dose distributions was calculated across the calculation matrix, and normalized to the local TG‐43 dose. For patient studies, DVH parameters (target and OARs) were also compared, including mean doses for 1 mm lung tissue shells created at 2 cm and 5 cm from target.

### Results

The phantom study indicates relative spatial differences between the two dose distributions, especially along the long axis of the source, where differences in excess of 40% can be observed. TG‐43 is shown to overestimate the dose in the vicinity of the source, and underestimate the dose relative to Acuros past an inflection point away from the source in lung tissue. In the plane perpendicular to the source, TG‐43 dose is 5% higher within 2 cm of the source. Similar spatial differences in the dose distribution are observable in the patient cases. Mean dose to medium in the lung shells indicate differences up to 5% higher (5 cm) and up to 2.2% lower (2 cm) than TG‐43. Absolute dose differences are minimal as indicated by DVH parameters similarity.

### Conclusion

TG‐43 and Acuros calculated dose distributions are spatially different. Since the absolute dose differences are minimal, their clinical significance remains to be further investigated.

**PO‐BPC‐Exhibit Hall‐14**

## Alignment of Multi‐Radiation Isocenters for Megavoltage Photon Beam

Y Zhang,^1*^ K Ding,^1^ G Cowan,^2^ E Armour,^1^ K Wang^1^

*Johns Hopkins University School of Medicine,^1^ Baltimore, MD, Elekta Oncology Systems,^2^ Atlanta, GA*

### Purpose

The accurate measurement of radiation isocenter (radiso) of a linear accelerator is critical and will impact the quality of radiation therapy. Traditional quality assurance (QA) procedure focuses on the measurement of single radiso, usually of 6X photon beam. Due to different flattening filters and steering parameters, the radiso of one energy mode can deviate from another if no special efforts were devoted during machine acceptance/commissioning phase. We presented a procedure of determining the multiradiso alignment of megavoltage (MV) with submillimeter accuracy using EPID‐based imaging method.

### Methods

An 8 mm ball bearing (BB) phantom was placed at the 6X radiso based on EPID images at various gantry and collimator angles. The 3D radiso shifts of other energy modes relative to 6X were determined. Beam profile scanning was used as an independent method to determine the 2D multiradiso alignment among flattened and flattening filter‐free (FFF) mode (6X, 10X, 15X, 6XFFF, and 10XFFF). To further quantify the impact of radiso offset on the target positioning accuracy, 10X radiso was manually offset from 6X by adjusting the bending magnet current, and the corresponding deviations of table rotation axis, aligned to 6X radiso, as well as the maximum deviation of the BB centroid during table rotation from the 10X radiso, were assessed.

### Results

From the EPID‐based method, we identified the 10XFFF was 0.96 mm/0.35 mm away from 6X radiso before/after tuning the beam steering parameter, which was confirmed by profile scanning method. The maximum deviation of BB phantom from 10X radiso was observed to linearly increase with the offset between 6X and 10X radiso; 1 mm 10X radiso offset translated to 1.8 mm deviation of BB phantom centroid (”target”) from the 10X radiso.

### Conclusion

We first investigated the relative multiradiso displacements of MV photon beams. The alignment of the multiradiso is particularly important for high‐precision radiotherapy techniques.

Garth Cowan is an employee of Elekta.

**PO‐BPC‐Exhibit Hall‐15**

## A Blueprint for Obtaining Intersocietal Accreditation Commission Dental Cone‐Beam CT Accreditation

M Wayson,^*^ M Arreola

*UF Health Shands, Gainesville, FL*

### Purpose

To provide the medical physics community with the experience of the University of Florida Health with obtaining dental cone‐beam CT (dCBCT) accreditation from the IAC.

### Methods

The CMS requires dentists to have advanced diagnostic imaging CT accreditation to claim Medicare reimbursements. Due to the infancy of dCBCT accreditation, it is important that experiences in this area are shared among the medical physics community. The IAC standards are generally divided between organizational and CT testing components. Of great interest to the medical physics community are the requirements for routing QC testing, which is addressed in IAC Accreditation Standards Part II, Section 2. The only test required is dose assessment, and no standards are given for any test. The units tested were the Kodak CS 9000 and 9300 and iCAT units. Manufacturer supplied phantoms and the Gammex 464 CT phantom were used for all testing.

### Results

The tests selected for annual evaluation were exposure linearity, slice thickness, artifact assessment, high contrast spatial resolution, image uniformity, and dose assessment (using both the AAPM Report No. 111 and less desirable CTDIvol methods). The most frequently used protocol was selected for each test. Exposure linearity had a COV less than 0.05 for all units. Minimum high‐contrast spatial resolution was 6 lp/cm. Pixel value deviation was no more than 166 from the center of a uniform image for all units. The greatest CTDIvol was 5.2 mGy, and the AAPM Report No. 111 method yielded point‐dose values close to the CTDIvol values obtained.

### Conclusion

UF Health used these tests for initial application for IAC accreditation for dCBCT, and other institutions may use this as a starting point for their own accreditation applications. As more institutions become accredited and dCBCT standards are set, a more unified approach to dCBCT accreditation will be established.

**PO‐BPC‐Exhibit Hall‐16**

## MR Imaging with DTI Can Predict Motor Outcome for Patients with Intracerebral Hemorrhage

M Kasam,^1^ S Konduru,^2^ R Konduru^2*^

*Mayo Clinic Rochester,^1^ Rochester, MN, University of Minnesota,^2^ Rochester, MN*

### Purpose

Intracerebral hemorrhage (ICH) is one of the major devastating forms of stroke. This disease currently does not have viable treatment options. Diffusion tensor imaging (DTI) records fractional anisotropy, which measures the degree of directionality in the diffusion process of water using a scale from 0 to 1, where 0 is equivalent to diffusion in all directions and 1 represents diffusion in a single direction and restriction in all others. Mean diffusivity measures the amount of diffusion. Disintegration of the CST is represented by a lower FA value and higher MD value.

### Methods

We have retrospectively collected multiple MRI scans of patients (with approved IRB protocol) admitted into our hospital from the past one year and have their MRIs between week 3, 6 after the occurrence of ICH with a DTI sequence on a 3‐Tesla GE scanner. We obtained FA and MD data on the cortical spinal tracts by drawing ROI on the cerebral peduncle, internal capsule, and pons of both ipsilateral and contralateral to the hematoma. The ROIs were used to calculate FA, MD, and volume. The correlation of FA, MD, and volume *s* were evaluated.

### Results

We obtained data on nine patients with basal ganglia hemorrhages. Preliminary analysis demonstrates that lower FA values correspond to worsening outcome and increased motor deficits. On week 3 and 6 FA of the cerebral peduncle on the affected side had direct correlation with worsening outcome and the correlation coefficients are 0.7 and 0.8, respectively. Additional data are needed to investigate further.

### Conclusion

ICH is a devastating disease with no approved medical therapies. These data may be an important biomarker that allows clinicians to monitor patient recovery and provide insight on the pathophysiologic course of this disease. When the pathophysiology is better understood, potential treatment can be tailored to those patients most likely to respond.

**PO‐BPC‐Exhibit Hall‐17**

## Application of MR Angiographic Fly‐Through Technique to Investigate the Femoral Vein Dimensions in PAD Patients

M Kasam,^1^ S Konduru,^2^ H Bektas,^3^ R Konduru^4*^

*Mayo Clinic Rochester,^1^ Rochester, MN, University of Minnesota,^2^ Rochester, MN, Ankara University,^3^ Ankara, Turkey, University of Rochester,^4^ Rochester, MN*

### Purpose

Development of the human vasculature database including femoral arterial parameters like maximum femoral artery diameter (MFAD), length, branching angles, etc., for both men and women will be critical for peripheral arterial disease (PAD) diagnosis for a treating clinician. Sporadic or limited work has been reported in clinical/preclinical research till date. This work was an important effort towards the development of human vasculature database using our newly developed method for estimation of stenosis‐Ref.

### Methods

Patient exams were performed under an IRB‐approved protocol. Raw MR angiographic images of 30 male and 30 female PAD patients were retrospectively processed and analyzed for MFAD. The measurement includes healthy, as well as symptomatic, regions of interest at different femoral arterial sections. MR angiographies with $1\;{\text{mm}}^{3}$ spatial resolution were used for this work. Clinical exams were acquired on 3.0 T GE scanners.

### Results

Data analysis: The angiogram was postprocessed using tree analysis and virtual endoscopy modules of Analyze 11.0 software to fly through the femoral artery. A statistical parameter called brightness area product (BAP) was defined as the sum of the intensities above the sample minimum intensity/threshold set by the user. This BAP is treated as a metric for the assessment of degree of stenosis. Different parameters were tested using two‐way Anova Student's *t*‐test.

### Results and Discussions

A statistical significance (p<0.05) has been noticed for MFAD and linear arterial measurements among men and women for both healthy and symptomatic sections of the peripheral artery. More work towards the development and validation of this study is under progress.

Reference: New Reconstruction Technique for MRA to “Fly Through” the Lumen of the Cerebral Vasculature Mallikarjunarao Kasam; Siva S Ketha; Soumya Konduru, ATVB 2013, FL, USA, Abstract 273.

**PO‐BPC‐Exhibit Hall‐18**

## Improved Commissioning Methods for Commissioning Linear Accelerators with Micro‐Multiple Leaves Collimator for Treating Stereotactic Radiosurgery

C Smith,^1*^ A Bacala,^1,2^ S Pella,^3^ T Leventouri^1^

*Florida Atlantic University,^1^ Boca Raton, FL, Department of Physics,^2^ Mindanao State University‐Iligan Institute of Technology, Iligan City, Philippines, South Florida Radiation Oncology,^3^ Boca Raton, FL*

### Purpose

Common methods for commissioning linear accelerators tend to neglect beam data for very small fields. An examination of the methods of beam data collection and modeling for comlinear accelerators revealed little to no discussion of the protocols for fields smaller than 4 cm×4 cm. This leads to decreased confidence levels in stereotactic radiotherapy (SRS). Accuracy in the calculations of the TPS is the foundation of high‐quality external‐beam radiation therapy outcome and improved patient care.

### Methods

AAPM TG‐155 is making several recommendations to improve the measuring process. These include the use of scanning water tank to increase the central alignment of the detectors, and using multiple detectors to confirm the accuracy of the measurements. Another area of improvement is replacing the protocol of using equivalent squares and interpolation to complete the output factor matrix with effectively measuring the output factor of each rectangular field. The output factors were completed by direct measurements only. Three areas of are addressed: detector alignment, actual measurements, and confirming the accuracy using a second type of detector. These improvements in the measurement process exhibit a marked improvement in the accuracy of the absolute dose calculated by the TPS.

### Results

The treatment planning system (TPS) is an essential tool in any radiation therapy treatment. The specific capabilities of one TPS to another may differ, but the fundamentals are the same. Applying the above methods improved the accuracy of the SRS plans by 18% in dose accuracy calculation to delivered.

### Conclusion

Improving the methods of collecting output factors led to an increase of confidence levels in the absolute dose delivered. Changes in the way we use the modern linear accelerator mean changes in the way we collect and commission the data. These methods improve the overall outcome, thus improving patient treatment outcome and care.

**PO‐BPC‐Exhibit Hall‐19**

## Dosimetric Impact of Roll Error on Lung Cancer Treatment Plan in Uniform Scanning Proton Therapy

S Rana^*^

*ProCure Treatment Centers, Oklahoma City, OK*

### Purpose

The purpose of this study is to investigate the dosimetric impact of roll error on the lung cancer treatment plan in uniform scanning proton therapy.

### Methods

Computed tomography (CT) dataset of lung cancer case treated with uniform scanning proton therapy was selected for this study. Proton plan for this case was generated using two fields. In order to observe the roll error on the dosimetric values, the CT dataset was resampled introducing various roll angles. A total of eight new CT dataset was generated for roll angles ranged from $-10^{{}^{\circ}}$ to $+10^{{}^{\circ}}$ with an increment of 2.5°. In the next step, eight new proton plans were generated based on the resampled CT dataset using the beam parameters identical to ones in the original proton plan. Dosimetric values from new proton plans were compared with that of original proton plan for the PTV coverage (dose received by 95% of PTV volume), maximum dose (Dmax) to the spinal cord, and the relative volume of total lung receiving at least 5, 10, and 20 CGE (V5, V10, and V20, respectively).

### Results

The relative difference (Δ) in the PTV coverage between the original and new proton plans ranged from −0.03% to −29.77%. For total lung, dosimetric values were always smaller for plans with roll angles $+2.5^{{}^{\circ}}$ to $+10^{{}^{\circ}}$, whereas the Δ ranged from −4.63% to +2.11% for roll angles $-2.5^{{}^{\circ}}$ to $-10^{{}^{\circ}}$. For the Dmax of the cord, the A ranged from −14.47% to +15.19%.

### Conclusion

The preliminary data from this study suggests that roll error of more than 5° could produce unacceptable decreased PTV coverage and increased Dmax to the cord for lung cancer in uniform scanning proton therapy.

**PO‐BPC‐Exhibit Hall‐20**

## Impact of Air Cavity to Critical Structures for Accelerated Partial Breast Irradiation (APBI) Brachytherapy

M Ashenafi,^*^ J Peng, J Harper, J Jenrette, K Vanek

*Medical University of South Carolina, Charleston, SC*

### Purpose

To investigate the feasibility of replanning due to air cavity effect for accelerated partial breast irradiation (APBI) brachytherapy.

### Methods

Dosimetric data of six patients treated with Contura multilumen balloon were retrospectively assessed for adaptive planning. Two axial CT image datasets were acquired; one was CT simulation used for planning and the other was prior to the first treatment. Ribs, skin (2 mm skin rendering), air cavity, and PTV_EVAL following NSABP B‐39 protocol were contoured on both CT datasets. Plans were generated using criteria including, for PTV_EVAL, V90PD (volume received 90% of prescribed dose (PD))>90%; V150PD<50cc; V200PD<10 cc. For skin and rib, Dmax (0.1 cc) (maximum dose)<125%PD. Dosimetric effect due to air cavity was evaluated by recomputing dose distributions on the second CT dataset by applying the same dwell planning time and comparing to the original plan. Differences were assessed for statistical significance (p<0.05) using Student's t‐test.

### Results

50% of patients had an average 1.4 cc air volume decrease between planning and treatment day. As a result, the average maximum skin dose increased by 18.7%. Similar dosimetry effect was discovered as the air cavity shown near the rib. The air volume decreased by 1.7 cc causing the maximums rib dose change by 15.5%. Overall, there was no statically significant difference in target dosimetery (V90PD, V150PD, and V200PD) due to change in air volume.

### Conclusion

Our study demonstrated a correlation between air volume change with skin and rib dose. Consideration of pretreatment 3D CT image acquisition and replanning is suggested when a significant change in air volume detected near critical structures.

**PO‐BPC‐Exhibit Hall‐21**

## Aperture Based Motion Management for Respiratory Gated VMAT SBRT: Improving Delivery

A Hsu,^*^ E Mok

*Stanford Cancer Center, Stanford, CA*

### Purpose

Accurate targeting of the tumor volume is crucial to the success of radiotherapy treatment and is of particular importance in hypofractionated treatments such as stereotactic body radiotherapy (SBRT). For many patients, especially those whose tumors exhibit motion due to respiration, alignment by orthogonal imaging and CBCT alone is not ideal. For these patients we use aperture gating on our TrueBeam sTx with Perfect Pitch couch. This work verifies the necessity of aperture‐based treatment.

### Methods

The TrueBeam STx with HD‐MLC and Perfect Pitch couch is ideal for treatments requiring a high degree of accuracy and precision. A 4D CT is used to determine the exhale phase and delineate the respective margins due to respiratory motion. Fiducial markers, such as gold seeds or stents, are used as a surrogate to determine the position of the PTV during treatment. A beam's‐eye‐view (BEV) aperture is created in a fluoroscopic setup field and used to determine the respiratory gating window at treatment. Initially, the patient is first aligned with orthogonal kV imaging. kV images are also acquired for during‐treatment verification. To verify the accuracy of this method, a plastic water phantom is placed on a moving platform to simulate the treatment. This method has been verified using EDR2 film and phantom measurement. Targets of various sizes were used.

### Results

During treatment, intrafraction kV images were taken at every beam on cycle for target positioning verification. Images can be taken at the beginning or at the end of the beam‐on 366 cycle. Film measurement confirmed the accuracy of the treatment to a target size as small as 1 cm diameter.

### Conclusions

The aperture‐gated method allows a potential reduction of the treatment margin to less than 5 mm for lung SBRT and for liver or pancreas SBRT a margin of 2 mm could be used.

**PO‐BPC‐Exhibit Hall‐22**

## Resting State Functional MRI Predicts Lesions when Diffusion Weighted Imaging (DWI) Fails in Ischemic Stroke

M Kasam^*^

*Mayo Clinic Rochester, Rochester, MN*

### Purpose

DWI is the current standard to identify and manage stroke. We assessed the hypothesis that resting state fMRI may detect the infarcts in patients with acute ischemic stroke more effectively than DWI.

### Methods

We studied ten patients within seven days of the acute onset of stroke patients retrospectively with an approved Institutional Review Board. All patients underwent multimodal MR imaging, including DWI and resting state fMRI sequences in the using a GE 3T MRI scanner. High resolution T2‐weighted images were also acquired. The resting state fMRIs were processed using a published method by a single rater with extensive experience in neuroimaging data processing to avoid interrater variability. DWI data was analyzed using Analyze 11.0 software.

### Results

fMRI provides more accurate information on the irreversible status of ischemic brain tissue during an acute stroke compared with diffusion weighted imaging. Similar results were observed across all the four patients studied.

### Conclusion

The results of this project have direct clinical implications. fMRI show areas of damage when DWI has normalized after an acute ischemic stroke. DWI can normalize after showing an area of restricted diffusion in areas of the brain that is irreversibly injured. Therefore, fMRI reliably indicates damaged brain tissue in the setting of acute ischemic stroke where DWI is unable to detect. Such information is critical to make treatment decisions when considering which acute stroke patients should undergo reperfusion therapies. fMRI provides complimentary information to CT/MRI perfusion and has the potential to obviate the need of CT/MRI perfusion which are invasive, and time and resource intensive. Our study is limited by their qualitative nature and small sample size.

**PO‐BPC‐Exhibit Hall‐23**

## Localization for H&N Patients who Undergo Rescans: A Case Study

L Courneyea,^*^ D Pafundi

*Mayo Clinic, Rochester, MN*

### Purpose

Study the necessity of creating new references images after a patient is resimulated.

### Methods

A retrospective case study was evaluated for the impact of forgoing new reference images for patient localization after a rescan. The patient studied underwent two rescans during a course of treatment. The first rescan was prompted by the removal of a Precise Bite from the patient's immobilization. The second rescan occurred after a new mask was created. To determine the impact of updating the reference images on the ability to accurately align the patient in an acceptable time, pretreatment CBCTs were matched to each of the three CT scans. The matches were performed using the automatching tools in Aria to a structure that contained the mandible, occipital bone, C1/C2, and C7/T1. The matches between the daily CBCTs and the three simulation scans were assessed for agreement of each of these structures using margins of 3 mm and 5 mm. The stability of the match was also tested by checking if the match changed when the algorithm was re‐run.

### Results

The accuracy of the automatch was strongly dependent on the CT set used. The structure which maintained the highest accuracy was C1/C2. When the CT and CBCT immobilization were the same, C1/C2 was matched to within 5 mm for 94.3% of the fractions. This decreased to 81% with a change in Precise Bite, 50% with a change in mask and 0%, if both items were changed. The stability of the match also decreased when changes were present.

### Conclusion

When a new mask is created, new images are essential for proper alignment. For the removal of a Precise Bite, the need for new images is dependent on site and the acceptable margin of error. Unstable matches were found to be an indication that new images are required.

**PO‐BPC‐Exhibit Hall‐24**

## Clinical Implementation of Intraoperative Electron Radiation Therapy (IOeRT)

T Amin, A Azzam, R Mahmood, Z Eltahir, H Selham, B Moftah, M Hussain^*^

*King Faisal Specialist Hospital & Research Center, Riyadh, Riyadh*

### Purpose

Intraoperative electron radiation therapy (IOeRT) is one of the developed technologies uses mobile linear accelerators for postresection patient treatment offers flexibility inside the operating room (OR). The overall implementation process of IOeRT is discussed here.

### Methods

The IOeRT delivering mobile linac equipped with electron energies 6, 9, and 12 MeV is warmed up with 500 MUs for each beam in the morning of the patient treatment. The dose output, energy ratio and some of the safety tests are performed by a radiation physicist. The IOeRT candidate patients are resected in OR followed by a possible single fraction electron dose to the tumor bed to treat residual disease.

### Results

Out of 11 patients treated to date, 30‐to‐75‐year‐old, ten male and one female, almost 75% will be receiving postsurgical chemotherapy. Along with pancreatic, retroperitoneal para‐aortic, urinary bladder treatment sites, almost half of the sites were portahepatis lymph nodes (LNs) and celiac LNs. A separate work is in process of compilation of pathological data. The single fraction doses ranging 10 Gy to 20 Gy were delivered using cones of mixed bevel angles 0°, 15°, and 30°, diameter ranging 6 cm to 10 cm with 0.5 cm bolus used in almost every treatment. The radiation therapy was delivered successfully as per clinical protocols, including AAPM TG‐72 guidelines. The pathological data analysis is in compilation stage for further analysis. The clinical outcomes would be presented separately after clinical follow‐up data are available in coming months.

### Conclusion

The IOeRT with high‐energy and high‐dose rate electron beams is a viable modality for pelvis and abdominal regions.

**PO‐BPC‐Exhibit Hall‐25**

## Skin Dose Measurements Using Optically Stimulated Dosimeters in High‐Dose‐Rate Surface Brachytherapy of the Breast

M Talmadge,^*^ I Iftimia

*Lahey Clinic, North Chelmsford, MA*

### Purpose

To experimentally verify skin‐to‐center dose (SCD) ratios using optically stimulated dosimeters (OSLD) to provide relative measurements in high‐dose‐rate surface brachytherapy of the breast.

### Methods

OSLDs calibrated using 6 MeV X‐rays were used to perform phantom‐based measurements with Accuboost applicators using an Iridium‐192 high‐dose‐rate source. Measurements were performed at the surface and center of phantoms of various thicknesses using several applicators in order to provide measured SCD ratios specific to each applicator and phantom thickness. Measured SCD ratios were then compared to vendor‐provided MCNP data. Additional measurements were made using a phantom to simulate a patient treatment in order to estimate the maximal SCD ratio.

### Results

Comparison of the experimental results for SCD ratio with those generated using MCNP produces good agreement. The results also provide estimates of the relative contribution to skin dose from orthogonal beam pairs allowing for the estimation of a net SCD ratio for the entire treatment when orthogonal beam pairs are used.

### Conclusion

These results provide experimental verification of the vendor‐provided SCD ratios and provide estimates of the maximum SCD ratios that can be expected when orthogonal beam pairs are used clinically.

**PO‐BPC‐Exhibit Hall‐26**

## Dosimetric Study of Two Rotational Intensity‐Modulated Radiotherapy in Thoracic Esophageal Carcinoma

R Zhang,^*^ W Bai, X Fan, R Qiu, D Liu, Z Chi, C Han

*Hebei Medical University Fourth Hospital, Shijiazhuang, Hebei*

### Purpose

To compare and analyze the dosimetry characteristics of volumetric‐modulated arc therapy (VMAT) versus constant dose rate intensity‐modulated arc therapy (IMAT) in the treatment of thoracic esophageal carcinoma.

### Methods

18 patients with thoracic esophageal carcinoma (the upper, the middle, and the lower thorax were six cases, respectively) were selected to design IMAT and VMAT treatment plans on Varian clinical 23IX conventional linear accelerator and Elekta Synergy new generation linac, respectively. Prescription dose of 60 Gy in 30 fractions, planning objectives for PTV were at least 95% reached the prescription dose and V110 no more than 10%. The maximum dose of spinal cord below 45 Gy and lung's V5≤55%, V20≤28%, V30≤18% were constrained. PTV and tissue dose‐volume parameters, machine MU, treatment time and γ pass rate were compared. Using SPSS 19.0 software paired t‐test analysis two sets of data.

### Results

IMAT group compared with VMAT group showing that PTV's CI increased by 4.6%; heart's V40, V45, V50; lung's V30 and treatment time were reduced by 11.1%, 11.3%, 8.4%; 5.6%; 38.1%, respectively. But spinal cord's D2, V40; lung's V3, V5, V10, V15, V20; and MU were increased by 2.9%, 47.7%; 7.1%, 8.3%, 7.8%, 8.3%, 8.5%; 48.8%, respectively. While there were no significant differences between IMAT and VMAT in the average dose of PTV, CTV, GTV, heart, spinal cord, lung and the γ (±3 mm, ±3%) pass rate, and DTA (±3 mm, ±3%) distance‐to‐agreement.

### Conclusion

IMAT in the treatment of time and tissue of high‐dose irradiated area is less than that of VMAT; but machine MU and tissue of low‐dose irradiated area is higher than that in VMAT. These two treatment methods can meet the clinical demand, can be selected according to the actual situation of the patient treatment.

The scientific research foundation of Hebei Provincial Health Department (project number: 20130253)

**Professional Symposium ‐ SAM Salon AB**

***Radiation Safety Officer Refresher***

**SA‐A‐Salon AB‐01**

## Radiation Safety Officer Refresher

D Broga,^1*^ B Blankenship^2*^

*VA Commonwealth University,^1^ Richmond, VA, Sharp Memorial Hospital,^2^ San Diego, CA*

This session will provide a refresher of RSO requirements and responsibilities under both regulatory and accrediting bodies. It will span radiation oncology, nuclear medicine, and radiology, as well as the expanding charges of the Radiation Safety Committee. In most hospitals, the RSO is a physician‐authorized user who relies heavily on the consultant medical physicist to supply guidance and analytic assessment to ensure regulatory compliance. Often the Medical Physicist may be asked to address radiation safety issues outside their normal scope of practice.

**Learning Objectives:**

1. Provide a broad overview of RSO responsibilities.
2. Identify the regulatory requirements applicable to a medical radiation protection program.
3. Discuss the expanding scope of the Radiation Safety Committees responsibilities under both regulatory and accrediting organizations.
4. Address the expanding radiation safety responsibilities for the consulting medical physicist.

Dean Broga (Imaging RSO Refresher)

Bette Blankenship (Therapy RSO Refresher)

**Therapy Symposium ‐ SAM Salon AB**

***Monte Carlo for Photon Treatment Planning; CV/SP and other Modern Algorithms; Monte Carlo for Electron Planning***

**SA‐B‐Salon AB‐01**

## Monte Carlo for Photon Treatment Planning; CV/SP and Other Modern Algorithms; Monte Carlo for Electron Planning

I Chetty,^1*^ M Miften,^2*^ J Cygler^3*^

*Henry Ford Health System,^1^ Detroit, MI, University of Colorado School of Medicine,^2^ Aurora, CO, The Ottawa Hospital Regional Cancer Ctr.,^3^ Ottawa, ON*

Treatment planning systems are indispensable for the radiation therapy treatment process. Despite the various versions implemented within different commercial systems, dose calculation algorithms generally share the same fundamental physics and can be separated into several categories. This session will review modern treatment planning algorithms, including convolution/superposition and Monte Carlo. For each algorithm, we will go through the physics behind the dose calculations and discuss the clinical implementation considerations. We will review their limitations and accuracies. We will discuss commissioning requirements for these algorithms and provide clinical examples with treatment plans.

**Learning Objectives:**

1. Understand Convolution/Superposition and other physics‐based algorithms for photon treatment planning.
2. Understand Monte Carlo dose calculation for photon treatment planning.
3. Understand Monte Carlo dose calculation for electron beams.
4. Know the commissioning requirements for the above algorithms.
5. Recognize their limitations and clinical applications.
6. Acknowledge the advantages and disadvantages for each algorithm.

Indrin Chetty (Monte Carlo Dose Calculation for Photon Treatment Planning)

Moyed Miften (Convolution/Superposition and other Physics‐Based Algorithms for Photon Treatment Planning)

Joanna Cygler (Monte Carlo Treatment Planning for Electron Beams)

**Diagnostic Symposium ‐ SAM Salon CD**

***Imaging Accreditation: RadSite, IAC***

**SA‐B‐Salon CD‐01**

## Imaging Accreditation: RadSite, IAC

P Patton^*^

*Orlando Regional Healthcare System, Windermere, FL*

This presentation briefly introduces RadSite to the medical physics community and explains our accreditation program that was approved by the Centers for Medicare and Medicaid Services (CMS) as meeting the requirements of the Medicare Improvements for Patients and Providers Act (MIPPA). The history of RadSite is reviewed, and the accreditation program requirements for imaging and safety policies, equipment, clinical examinations, and physics testing are explained. Special attention will be given to the physics testing requirements portions of the accreditation standards.

**Learning Objectives:**

1. Review the RadSite accreditation process.
2. Review clinical requirements of the accreditation process.
3. Discuss the Medical Physicists role and responsibilities for RadSite accredited facilities.

**SA‐B‐Salon CD‐02**

## Imaging Accreditation: RadSite, IAC

S Balter

*Columbia University Medical Center, New York, NY*

This presentation briefly introduces carotid artery stenting (CAS) and the IAC. Carotid artery disease causes about half of all strokes. The two major invasive treatments are surgery or stenting. CAS is performed using an interventional fluoroscope. The goal of IAC in this domain is to facilitate optimizing the safety and efficacy of CAS. Medical physicists play a key role both in image optimization and in radiation management. The standard includes requirements for equipment, safety, and physics testing. Data are required for both the initial application and for periodic quality management. The standard's requirements exceed those needed to minimally comply with most radiation regulations. One goal of IAC is to integrate QMPs into the CAS environment as active contributors. The presentation will review relevant portions of the standard, and provide a sampling of topics that the QMP might consider implementing in this role.

**Learning Objectives:**

1. Review the IAC accreditation process.
2. Review clinical carotid stenting procedures.
3. Outline relevant Medical Physics processes and responsibilities.
4. Outline physics and related requirements for a carotid stenting program.

**Therapy Symposium ‐ SAM Salon AB**

***Plan Quality: Analyzing and Quantifying Plan Quality as a Key Component of a Total Quality System***

**SA‐C‐Salon AB‐01**

## Plan Quality: Analyzing and Quantifying Plan Quality as a Key Component of a Total Quality System

B Nelms,^1*^ R McInturf,^2*^ V Feygelman,^3*^ G Robinson^4*^

*Merrimac,^1^ WI, Cancer Care Centers of South Texas,^2^ San Antonio, TX, H. Lee Moffitt Cancer Center,^3^ Tampa, FL, Radiation Oncology Resources,^4^ Goshen, IN*

In the medical physics realm, the mention of “quality assurance” (QA) is often associated with topics such as system commissioning or per‐patient verification of TPS dose calculations and machine delivery. However, in reality, a total quality system has many more components. One vital upstream component is the quantification and assessment of the actual “plan quality” (i.e., the abilities of the TPS, modality, and treatment planner to create high‐quality plans that meet clinical needs and are robust with respect to potential errors). The quantification and study of plan quality across many TPS, modalities, and treatment planners has been the major point of study for a group of clinicians and researchers for the past few years. They will describe the methods, experiences, and lessons learned. Scientific studies of plan quality variation (and the root causes) will be presented, and a dedicated “Plan Challenge” on a challenging SBRT case will be performed just for this meeting. Another topic will introduce plan quality measures as a key component in a Maintenance of Certification (MOC) project at a large clinic. Yet another talk will concentrate on the role of plan quality measures in the evolving recommendations for TPS commissioning and will provide a glimpse into the upcoming TG 244 report. The session will close with discussions of practical implementation, automation, and application to commissioning new autoplanning software tools, plus other future directions to further improve radiation plan quality and safety going forward.

**Learning Objectives:**

1. To learn the history and methodology to create comprehensive and customizable plan quality algorithms.
2. To learn the measured variations in plan quality based on: treatment planner, TPS, and delivery modality.
3. To understand the difficulty to achieve any/all physician's objectives prior to planning (but postcontouring) and realistic expectations prior to plan optimization. To learn how to do patient‐specific adjustments, or normalizations, of plan quality scores.
4. To learn a comprehensive plan quality metrics for a MOC project.
5. To discuss standard sets of clinical plans (patient data) for TPS QA along with a clear list of aggressive, multicriteria plan objectives, with the goal to be to quantify and benchmark.
6. To learn the results of the Plan Challenge planned and dedicated specifically for this AAPM meeting.

Benjamin Edward Nelms (Introducing the ‘Plan Quality Algorithm’ as a method to customize and automate the quantification of plan quality)

Benjamin Edward Nelms (Introducing the ‘Plan Quality Algorithm’ as a method to customize and automate the quantification of plan quality)

Rebecca McInturf (Using Plan Quality Metrics for Maintenance of Certification (MOC))

Vladimir Feygelman (Integrating Plan Quality Metrics into Comprehensive TPS Commissioning:

A Look Ahead to TG 244)

Greg Robinson (Results of the 2014 AAPM Spring Meeting Plan Challenge [SBRT])

**Diagnostic Symposium ‐ SAM Salon CD**

***CT IAC Accreditation / Clinical CT for Physicists***

**SA‐C‐Salon CD‐01**

## CT IAC Accreditation / Clinical CT for Physicists

R Pizzutiello,^1*^ J Siegelman^2*^

*Upstate Medical Physics,^1^ Victor, NY, Brigham and Women 's Hospital,^2^ Boston, MA*

With the proliferation of CT scanners and increasing interest in accreditation, many physicists are being asked to support facilities seeking IAC CT Accreditation. IAC CT Accreditation is particularly attractive to facilities utilizing small field of view cone‐beam CT (CBCT), such as ENT and dental practices, since these scanners cannot image larger size phantoms required for ACR Accreditation. This presentation addresses the specific IAC requirements for medical physicists supporting sites seeking IAC Accreditation. Some of these requirements are unique to IAC CT Accreditation, and will be addressed in detail. Common weaknesses in facility submissions relating to medical physics services will be presented. An overview of the IAC organization, Standards and the actual process of becoming accredited will also be presented. The relationship between the medical physicist and radiologist has grown in importance in the present regulatory environment, and is advocated for in the recently released *AAPM Medical Physics Practice Guideline 1.a: CT Protocol Management and Review Practice Guideline*. Collaborative patient image review is an important mechanism to facilitate dialogue about dose optimization and clinical requirements. Clinically significant diagnostic challenges with medical vignettes and patient images will be presented. This session will include details surrounding an important accreditation body and modality, and critical considerations in the ever‐developing paradigm of the patient dose and image quality continuum with respect to collaboration between clinicians and medical physicists.

**Learning Objectives:**

1. Review the IAC CT Accreditation program and typical facilities seeking accreditation.
2. Review relevant Medical Physics processes and responsibilities unique to IAC CT Accreditation.
3. Review common problems with medical physics reports submitted to IAC CT Accreditation.
4. Review the process of applying for IAC CT Accreditation.
5. Observe pathology‐mimicking artifacts in head CT.
6. Review image characteristics necessary for accurate diagnoses in spine CT.
7. Review expectations for screening, nonscreening, and specialty chest CT.
8. Observe image features that create challenges for lesion detection in liver CT.

Robert Pizzutiello (Understanding IAC CT Accreditation)

Jenifer Siegelman (Facilitating Communication for Dose Optimization in CT)

**Therapy Symposium Salon AB**

***Image Registration for Radiation Therapy Applications***

**SA‐D‐Salon AB‐01**

## Image Registration for Radiation Therapy Applications

D Wang,^1*^ P Balter^2*^

*University of Iowa,^1^ Iowa City, IA, UT MD Anderson Cancer Center,^2^ Houston, TX*

Image registration is an indispensable process for radiation therapy, involved in both treatment planning and image guidance at treatment. For treatment planning applications, this session will review multimodality image registrations between treatment planning CT and 4D CT, MRI, PET image set, as well as CT and dose map from previous treatment, if available. For each modality, we will discuss the clinical implementation considerations. We will also discuss deformable image registrations. For image‐guidance applications, we will discuss registration between CBCT and treatment planning CT image sets. We will review the limitations and accuracies of the registrations. We will discuss the clinical considerations of selection of region of interest (ROI) and provide some clinical examples. We will also discuss the compromises needed for daily IGRT and how these are communicated among the treatment team.

**Learning Objectives:**

1. Understand multimodality image registration for treatment planning applications.
2. Learn clinical implementation considerations of image registration for treatment planning applications.
3. Understand accuracy and limitations of deformable image registrations.
4. Understand the registration between image‐guidance image set and treatment planning CT.
5. Recognize the limitations and clinical applications of image‐guidance registration.
6. Acknowledge the advantages and disadvantages for selection of ROI.
7. Discuss requirements of communication and means of communication among the treatment tam for IGRT.

Dongxu Wang (Image Registration for Treatment Planning)

Peter Balter (Image Registration for IGRT)

**Diagnostic Symposium ‐ SAM Salon CD**

***MR Safety / The New MR ACR QC Manual***

**SA‐D‐Salon CD‐01**

## MR Safety / The New MR ACR QC Manual

R Pooley,^1*^ J Felmlee^2*^

*Mayo Clinic,^1^ Jacksonville, FL, Mayo Clinic,^2^ Rochester, MN*

Clinical diagnostic medical physicists may be responsible for implementing and maintaining a comprehensive MR safety program. Hospitals and clinics will look to the physicist to not only understand guidelines, regulations, and accreditation requirements related to MR safety, but also to understand details related to patient care and employee safety. Accrediting bodies including the ACR, IAC, RadSite, and the Joint Commission each include aspects of MR safety into their imaging accreditation programs, and MIPPA regulations further raise the significance of noncompliance. In addition, the Joint Commission recently announced New and Revised Diagnostic Imaging Standards for accredited health‐care organizations which include aspects of MR Safety. The clinical medical physicist must be aware of all of these requirements, which will be covered in this presentation. In addition to regulations and requirements, the clinical medical physicist must be aware of practical aspects of MR safety. These include issues of direct patient care and employee safety. How does one set up and maintain a safety program? How can proper site planning lead to improved MR safety? How can ferromagnetic detectors be used to enhance the screening process? And what can be done when faced with a new implanted medical device for which the patient has no card? This presentation will also review practical patient care and employee safety aspects of MR safety.

**Learning Objectives:**

1. Understand requirements and recommendations related to MR safety from accrediting bodies and federal regulations.
2. Identify best practices for dealing with implanted devices.
3. Review aspects of MR safety involved in site planning.
4. Identify use and limitations of ferromagnetic detectors for patient screening.

Robert Pooley (MR Safety: Requirements and Practical Aspects)

Joel Felmlee (MR Safety: Requirements and Practical Aspects)

**SA‐D‐Salon CD‐02**

## MR Safety / The New MR ACR QC Manual

R Price

*Vanderbilt Medical Center, Nashville, TN*

The planned publication date of the revised ACR *MRI Quality Control Manual* is the first or second quarter of 2014. The manual will be made available in electronic form on the ACR website. The website will include a link to frequently asked questions (FAQs), as well as planned annual updates to the manual. The required performance parameters identified in the 2004 version of the manual have for the most part not changed. An exception is that the previously required test for RF “cross‐talk” has been removed and is now listed as optional. As part of the revised annual performance evaluation, the qualified medical physicist/MR scientist must: (1) repeat and verify the weekly technologist's QC measurements, (2) perform the scans required for accreditation submission and evaluate those measurements with respect to the criteria specified in the most recent version of ACR Phantom Test Guidance Document, and (3) assess the site's MRI safety program as recommended by the “ACR Guidance Document for Safe MR Practices: 2013”.^(1)^ The safety assessment should include a review of written policies, signage and safety training. The revised manual also includes the description and suggested use of the ACR “Small Phantom” for extremity or other special‐purpose MRI systems that may not be able to accommodate the original large phantom. The revised manual includes alternate test procedures for both SNR and magnetic field homogeneity as described in the National Electrical Manufacturers Association (NEMA) publications and in AAPM Report 100. The alternate methods include both a single‐image method and a two‐image method for SNR assessment in addition to the original method recommended in the 2004 ACR manual. Alternate test methods for magnetic field homogeneity include the “phase‐map” method and the “bandwidth‐difference” method (Chen HH, et al Medical Physics 2006; 33:4299‐4306). The bandwidth‐difference method can be used in systems that do not provide access to either phase images or spectroscopy. Not included in the revised manual is a specific method for testing multi‐element array coils or for testing parallel imaging with acceleration. The current manual recommendation is that the maximum SNR be measured for each individual coil element in a manner similar to surface coil SNR assessment. It is anticipated that when the most appropriate method for testing multi‐element arrays has been identified and approved, the method will be incorporated into the manual by means of the annual update. All discussed revisions should be considered to be preliminary until final approval has been granted by the American College of Radiology.

**Learning Objectives:**

1. Identify changes in the Technologist's Section
2. Review recommendations for the required annual site safety program assessment
3. Review the revised set of tests for annual performance testing
4. Present details of the alternate tests for SNR and field homogeneity

1. Kanal E, et al. JMRI. 2013; 37:501–530.

**SUNDAY, MARCH 16**

**Therapy Symposium ‐ SAM Salon AB**

***Safety 1: Incident Learning Systems in Radiation Therapy***

**SU‐A‐Salon AB‐01**

## Safety 1: Incident Learning Systems in Radiation Therapy

E Ford,^1*^ G Ezzell,^2*^ D Gilley^3*^

*University of Washington,^1^ Seattle, WA, Mayo Clinic Arizona,^2^ Phoenix, AZ, AAPM,^3^ College Park, MD*

Identifying and analyzing safety‐related events is a proven way to enhance the quality and safety of care. Data demonstrate that patient outcomes are improved when health‐care providers actively engage in incident reporting. This session will review existing data and discuss how incident learning systems are currently used in radiation oncology. The essential features of incidents and near‐misses will be presented, along with the criteria for reportable events under the various applicable regulations. The use of incident learning system at the departmental level will be discussed, along with recommendations for structuring and operating such systems. Though incident learning is coming into wider use within clinics, there is still a major unmet need to collect and share such information between clinics. This session will highlight one new system designed to enable this: the national Radiation Oncology Incident Learning System (RO‐ILS). This system, sponsored by ASTRO and AAPM, provides a means for sharing safety improvement information with the legal protections afforded by its status as a Patient Safety Organization. Early experiences with this system will be shared, along with experiences from the SAFRON system, an internationally used open‐incident reporting system.

**Learning Objectives:**

1. Understand the definitions of events and near‐misses and how to structure an incident learning system at the department level.
2. Learn about the national effort to establish the national Radiation Oncology‐Incident Learning System (RO‐ILS).
3. Understand the investigation process and lessons learned with SAFRON, an international incident learning system in radiation oncology.

Eric Ford (Using Incident Learning Systems to Improve Patient Safety: A Clinical Perspective) Gary Ezzell (Creation of a National Incident Learning System to Improve Patient Safety) Debbie Gilley (Lessons learned from SAFRON: Using Incident Learning in Multi‐Institutional Settings)

**Mammography Symposium Salon CD**

***Advances in Breast Imaging***

**SU‐A‐Salon CD‐01**

## Mammography Symposium Salon CD *Advances in Breast Imaging* Advances in Breast Imaging

J Lewin,^1*^ C Hruska^2*^

*Diversified Radiology of Colorado,^1^ Mayo Clinic,^2^ Rochester, MN*

**Advanced Applications of Digital Mammography: Tomosynthesis and Contrast‐enhanced Digital Mammography** ‐ John Lewin, MD., FACRC

Clinical studies of digital mammography versus screen‐film mammography showed limited benefits in performance for digital over screen‐film leading many to conclude that the true benefit of digital mammography would be in the advanced applications made possible by that technology. Now two of those applications, digital breast tomosynthesis (DBT) and contrast‐enhanced digital mammography (CEDM), have advanced to the stage of clinical use. Both technologies show clear performance advantages over standard mammography, but both have their limitations in terms of cost and, in the case of CEDM, the need to inject IV contrast. This talk will review the both DBT and CEDM, including the technology, clinical evidence, current clinical status, and recent technological developments.

**What Medical Physicists Need to Know about Breast Imaging with Nuclear Medicine Technology** ‐ Carrie Hruska, PhD.

The use of dedicated nuclear medicine technologies for breast imaging, such as molecular breast imaging (MBI), breast specific gamma imaging (BSGI), positron emission mammography (PEM), and dedicated breast PET, is growing and may be coming to your site. Will you be ready? Although these technologies have been under study for over a decade, concerns about radiation dose and unclear clinical indications for their use have limited widespread acceptance. Today, improved detector technology and modified imaging protocols permit imaging at reduced radiation doses. A growing body of clinical data supports their use as a functional complement to mammography and a low‐cost alternative to MRI in certain diagnostic and screening settings.

**Learning Objectives:**

1. Give participants an overview of the various nuclear medicine technologies for breast imaging.
2. Demonstrate how each technology is being used in clinical practice and research.
3. Enable participants to discuss radiation doses used in breast imaging and their associated risk.

John Lewin (Advanced Applications of Digital Mammography: Tomosynthesis and Contrast‐enhanced Digital Mammography)

Carrie Hruska (What Medical Physicists Need to Know about Breast Imaging with Nuclear Medicine Technology)

**Therapy Symposium Salon AB**

***Safety 2: Hands‐On Session: FMEA in Safety***

**SU‐B‐Salon AB‐01**

## Safety 2: Hands‐On Session: FMEA in Safety

J L Johnson

*UT MD Anderson Cancer Center, Houston, TX*

Medical physicists are responsible for providing clinical service to patients undergoing diagnosis and treatment and are delegated the primary caretakers of safety. As the complexity of the technology in providing these services has increased, so have the risks to the patient, the healthcare workers, and the public. Medical physicists' unique knowledge and skills must expand on what they already know and do with the complexity of technology to minimize familiar and unexpected risks as much as possible. One way to broaden their safety knowledge and skills is to formalize their approach through use of failure mode and effects analysis (FMEA). This hands‐on session will show that FMEA is simply a more systematic approach to what medical physicists currently do in identifying risks for a clinical service process. Participants will complete a process FMEA on a single process step of a 3D conformal radiotherapy (3D CRT) breast treatment case.

**Learning Objectives:**

1. Describe process failure mode and effects analysis (FMEA) concepts.
2. Apply FMEA on a single process step in a 3D‐CRT case.

**SU‐B‐Salon AB‐02**

## Safety 2: Hands‐On Session: FMEA in Safety

E Yorke,^*^ A Houston, A Kelly, H Piszko, N Stein, J Topf

*Memorial Sloan‐Kettering Cancer Center, New York, NY*

Failure Modes and Effects Analysis (FMEA) is a quality management tool adopted from industry and introduced to radiation oncology a few years ago. Together with other QM measures—incident reporting and fault‐tree analysis — FMEA helps to identify risky points in clinical processes and to devise error (or ‘failure mode’) mitigation measures. FMEA has been thoroughly described in several publications, including the Proceedings of the 2013 AAPM Summer School. Typically, a group of involved clinicians choose a process, list its steps (process tree), and then brainstorm as to what failure modes at each step can lead to risky situations. They then rank each failure mode (1‐10, with 10 being the worst) as to its occurrence likelihood (O), potential severity (S), and likelihood of detection before reaching the patient (D). Its potential impact is summarized by the Risk Probability Number (RPN), the product of O, S, and D. FMEA is iterative, as mitigation strategies need to be reevaluated after implementation, and increased experience may bring new failure modes to light. FMEA is well‐suited to both large and small‐scale clinical processes. Small‐scale processes, with a limited number of steps and a small number of participants, can be performed quite quickly. This lecture presents two examples of this sort. The first is an FMEA of monitor‐unit calculations for simple, after‐hours (‘on‐call’) treatments. The FMEA was performed by one physicist and, through informal presentations to other physicists and periodic therapist and new resident in‐services, has helped modify department policy over a several years. The second is a newly initiated FMEA of deep‐inspiration breath‐hold for treatment of the left breast, performed by the physicists and simulation therapists involved in the treatment. We expect this FMEA to help identify risky areas and guide development of mitigating measures for this process.

**Learning Objectives:**

1. To know the basic steps of an FMEA.
2. To know the definitions and relative scales for the FMEA parameters O, S, and D.
3. To understand the FMEA approach in relation to small‐scale clinical processes.

**Mammography Symposium ‐ SAM Salon CD**

***Stereotactic Breast Biopsy/MRI/Ultrasound***

**SU‐B‐Salon CD‐01**

## Stereotactic Breast Biopsy/MRI/Ultrasound

W Geiser,^*^ D Reeve^*^

*UT MD Anderson Cancer Center, Houston, TX*

**BICOE Stereotactic Breast Biopsy and Ultrasound** ‐ William Geiser, MS, DABR

In October 2007, the American College of Radiology started a new program called the Breast Imaging Center of Excellence (BICOE). The BICOE designation is awarded to breast imaging centers that have earned accreditation in the ACR's voluntary breast‐imaging accreditation programs, including breast ultrasound with ultrasound guided biopsy and stereotactic breast biopsy, as well as the mandatory mammography accreditation program. Significant changes have been made to the program requirements for breast ultrasound that will need to be implemented by June of 2014 in order obtain accreditation. Along with the prone stereotactic breast biopsy systems, several mammography systems have add‐on stereotactic biopsy systems that will need to be accredited if the facility wishes to obtain the BICOE designation. This lecture is designed to give the medical physicist the information needed to perform the necessary quality control testing on stereotactic breast biopsy, tomosynthesis‐guided biopsy, and breast ultrasound systems, to obtain accreditation for those modalities. With this information the medical physicist will be able to help the facilities that they work with obtain the designation of Breast Imaging Center of Excellence.

**Learning Objectives:**

1. Understand the annual test requirements for stereotactic breast biopsy systems accredited by the American College of Radiology.
2. Understand the testing requirements for breast ultrasound systems in the ACR BICOE program.
3. Help facilities obtain the designation of Breast Imaging Center of Excellence.

**Breast MR Imaging and Quality Control** ‐ Donna M. Reeve, MS, DABR, DABMP

MR imaging of the breast is an important adjunct to mammography and ultrasound for the detection of breast cancer and for breast biopsy guidance. Diagnostic breast MRI protocols typically include T2‐weighted fat saturated images and 3D T1‐weighted dynamic contrast enhanced (DCE) multiphase images using a Gadolinium‐based contrast agent. The DCE images are used to determine the kinetic signature of enhancing tissues in order to differentiate between benign and malignant lesions. MR spectroscopy and diffusion weighted images (DWI) may also be acquired in order to provide additional diagnostic information. The diagnostic value of breast MRI scans can be improved by choosing acquisition parameters that optimize image quality parameters such as SNR, contrast, and spatial resolution. As with any MRI protocol, achieving the desired image quality must balance the need for acceptable scan times. The timing and temporal resolution of the DCE series are important considerations. Breast radiofrequency (RF) coils are phased array coils capable of simultaneous bilateral imaging. A comprehensive breast MRI quality control program is important to assess the performance of the MRI system, as well as the breast RF coils.

**Learning Objectives:**

1. Provide and overview of breast MR imaging and MR‐guided biopsy procedures.
2. Describe breast MR image quality criteria and protocol optimization.
3. Discuss the components of a breast MRI quality control program.

William Robert Geiser (BICOE Accreditation of Stereotactic Breast Biopsy and Breast Ultrasound Systems)

Donna Reeve (Breast MR Imaging and Quality Control)

**Professional Symposium ‐ SAM Salon AB**

***Error Prevention***

**SU‐C‐Salon AB‐01**

## Taking Responsibility for Safety

G Sherouse

*Landauer Medical Physics, Charlotte, NC*

Much has been made of the need for robust clinical processes in support of safety and quality management, and that is indeed a crucial element of practice in which the medical physicist typically plays a key role as the only expert in the organization with any formal engineering training. Equally critical is attention to safety in the design of medical devices and in the superdevices created when they are interconnected and interact. The role of the clinical medical physicist in promoting safety of device design is indirect, but the local implementation of any new or repurposed technology should always include a careful analysis of hazards that device design might present in a given milieu, and again it is the clinical medical physicist who plays a leadership role in performing the hazard analysis and mitigation. This presentation will focus on neither of those essential roles of the medical physicist, which get good attention elsewhere, but rather goes further into the uncomfortable territory of professional responsibility. The very heterogeneity of backgrounds and training that many tout as the long‐standing strength of legacy pathways into medical physics have also tended to foster a wide diversity in the ways that both medical physicists and others view the standing, role, and responsibilities of the clinical medical physicist. The technology for which we take responsibility is complex and inherently life‐threatening. It is incumbent on medical physicists to establish themselves locally as the professionals who manage reliability, to take that responsibility seriously in our training and our practice, and to always be prepared to do what must be done to protect our patients from harm. We will survey some case studies of medical radiation accidents in which patients have been harmed or killed, with a particular emphasis on the ways in which clinical medical physicists have performed exceptionally (on either side of the bell curve). Some discussion will be offered as to the specific ways in which medical physicists should think about their role and responsibilities in the provision of highly reliable care.

**Learning Objectives:**

1. To better understand that the medical use of radiation is inherently dangerous.
2. To understand the role medical physicists have played in some high‐profile radiation accidents.
3. To review the professional role and responsibilities of the clinical medical physicist in the safety of clinical practice.

**SU‐C‐Salon AB‐02**

## A System‐Theoretic Approach to Safety in Radiotherapy

R Reddick

*Varian Medical Systems, Las Vegas, NV*

Sophisticated techniques and technologies have now become the standard of care in radiation oncology. Complexity has increased the demands on radiotherapy professionals and equipment manufacturers for assuring safe operation. The keys to radiotherapy safety are no longer found only in device reliability, training, traditional QA, and vigilance. A broader, system‐wide view must be adopted, where the “system” includes the entire constellation of devices, processes, and persons. Risk analysis must not only look at the interactions among the medical devices and their operators, but also at the effects of workflows, policies, regulations, environmental disturbances, and even culture. Preventing accidents is not as much an error‐prevention problem as it is a risk control problem — controlling the risk of harm when an error inevitably occurs. We present a system‐theoretic approach to risk assessment and safety design in radiotherapy, and use actual radiotherapy incidents to illustrate the concepts.

**Learning Objectives:**

1. Understand how system safety concepts apply to radiotherapy.
2. Learn the basic concepts of system‐theoretic hazard analysis.
3. Appreciate how system safety concepts manifest in actual radiotherapy incidents.

**Professional Symposium ‐ Interactive Salon CD**

***Practice Guidelines***

**SU‐C‐Salon CD‐01**

## Practice Guidelines

J Seibert,^1*^ L Fong de los Santos,^2*^ J Smilowitz^3*^

*UC Davis Medical Center,^1^ Sacramento, CA, Mayo Clinic,^2^ Rochester, MN, University of Wisconsin,^3^ Madison, WI*

Recently, the American Association of Physicists in Medicine (AAPM) established a Medical Physics Practice Guidelines (MPPG) initiative to provide a clear and concise description of the minimum level of medical physics support that the AAPM would consider to be prudent in all clinical practice settings. As accreditation of clinical practices becomes more common, MPPGs will be crucial to ensuring a consistent benchmark for accreditation programs. MPPG reports will be freely available to the general public. Accrediting organizations, regulatory agencies, and legislators will be encouraged to reference these MPPGs when defining their respective requirements. Support includes, but is not limited to, staffing, equipment, machine access, and training. This session will describe the purpose and scope of MPPGs, the procedure for the development of a MPPG, and reports from TG‐230 MPPG #3 *Development, Implementation, Use and Maintenance of Safety Checklists*, TG‐243 MPPG #4 *Levels of Supervision for Medical Physicists in Clinical Training*, and TG‐244 MPPG #5 *Commissioning and QA of External Beam Treatment Planning System Dose Calculations*.

**Learning Objectives:**

1. Understand the concept, scope, and process of MPPG from the AAPM.
2. Understand the goals, methodology, and recommendations of TG‐230 MPPG #3.
3. Understand the goals, methodology, and recommendations of TG‐243 MPPG #4.
4. Understand the goals, methodology, and recommendations of TG‐244 MPPG #5.

J. Anthony Seibert (Levels of Supervision for Medical Physicists in Clinical Training)

Luis Fong de los Santos (Development, Implementation, Use and Maintenance of Safety Checklists)

Jennifer Smilowitz (Commissioning and QA of External Beam Treatment Planning System Dose Calculations)

**Therapy Symposium ‐ SAM Salon AB**

***Safety 3: Radiation Oncology Safety Stakeholders***

**SU‐D‐Salon AB‐01**

## Safety 3: Radiation Oncology Safety Stakeholders

B Fraass,^1*^ J Schewe,^2*^ C Negrut^3*^

*Cedars‐Sinai Medical Center,^1^ Los Angeles, CA, Philips,^2^ Fitchburg, WI, Accuray,^3^ Sunnyvale, CA*

In recent years there has been an increased interest in patient safety in Radiation Oncology, in part due to highly‐publicized errors, such as those described in the New York Times. Clinical practitioners, professional organizations, manufacturers, and regulatory agencies have each responded to safety concerns in their own way, generating white papers, task group reports, new product safety features, regulatory changes, and an increased emphasis on training and credentialing. There has also been an increased interest in the use of techniques related to Risk Management, Human Factors, and Usability Engineering, both in industry and in the clinic. In particular, the TG‐100 Report recommends the use of risk management tools to identify and mitigate safety issues in clinical practice. In addition to each group working independently, there has also been an increased interest in stakeholders from all areas working together in a coordinated way. The Radiation Oncology Safety Stakeholders Initiative (ROSSI) was created in 2010 by members of AAPM, ASTRO, and the RT Industry, with a mission “to recommend and facilitate safety improvements in radiotherapy through a common, independent and impartial vision broadly based on diversity of experience and knowledge among radiation oncology professionals.” The first speaker in this session will provide a summary of the history, goals, structure, progress to date, and future directions of the ROSSI collaboration. The second speaker will describe the role of Safety Risk Management in the development and support of medical devices, including a discussion of the similarities and differences in its use in industrial and clinical settings. The final speaker will give an overview of Usability and Human Factors issues in medical software, along with a review of recommendations from the ROSSI working groups on Usability and Error Messages.

**Learning Objectives:**

1. Understand the structure and mission of the Radiation Oncology Safety Stakeholders' Initiative (ROSSI).
2. Learn about how the clinical community and vendors are collaborating to help improve the usability, quality, and safety of medical devices and clinical practice.
3. Understand the basics of Safety Risk Management, its relationship to the product life cycle, and the similarities and differences in how it applies to products and clinical practice.
4. Understand the basics of Usability and its relationship to the safety of medical devices, including problems and recommended improvements with the content and frequency of software error messages.

Benedick Fraass (An Overview of the Radiation Oncology Safety Stakeholders Initiative)

James Schewe (Safety Risk Management in Radiation Therapy: A Software Manufacturer Perspective)

Cristina Negrut (Safety in Radiation Therapy: Usability from a Software Engineering Perspective)

**Mammography Symposium ‐ SAM Salon CD**

***MQSA/ACR Update***

**SU‐D‐Salon CD‐01**

## MQSA/ACR Update

E Berns,^1*^ M Seddon^2*^

*University of Colorado Health Science,^1^ Denver, CO, Florida Hospital,^2^ Altamonte Springs, FL*

**ACR/MQSA Update** ‐ Eric Berns, PhD, DABR

Digital mammography has become the technology of choice for breast imaging with many FDA‐approved systems already available and more on the way. Currently the FDA requires facilities to use quality control manuals published by the FFDM unit manufacturer. There are several accrediting bodies that run programs approved by the FDA. This lecture will provide updates on the FDA activities, ACR requirements, the upcoming ACR DM QC Manual for digital mammography. Approval and accreditation processes for digital breast tomosynthesis will also be introduced.

**Learning Objectives:**

1. Understand the requirements for accrediting FFDM systems.
2. Understand the requirements for certifying tomosynthesis systems with the FDA.
3. Be able to navigate the ACR and FDA websites to find required information and documents for certification and accreditation.

**Advances in Full Field Digital Mammography** ‐ Mark Seddon, MS DABR

In 2011 the FDA switched form a pre‐market approval (PMA) process to approve full‐field digital mammography (FFDM) systems to their 510k‐approval process. Since that time 19 new systems or variations of existing systems have been approved full‐field digital mammography. These systems include computed radiography systems, full‐field detector systems with new and existing detector technologies, and a photon counting system. At the time of the switch there were five vendors with approved systems; there are now 13 vendors in the FFDM market. Tomosynthesis and 2D synthesized images for interpretation have also been approved for screening mammography. This lecture will give the medical physicist information on the systems approved since 2011. Included will be information on detector technology, some of the differences in QC for the different vendors, and some of the new technologies that are being used to lower dose for patient imaging.

**Learning Objectives:**

1. Understand the new detector technologies since 510k.
2. Be able to find information needed to perform acceptance testing and continuing QC on the new systems.
3. Understand some of the new technologies being employed to lower patient dose in FFDM.

Eric Berns (ACR Update on FFDM Accreditation)

Mark Seddon (Update in FFDM since 510k)

MONDAY, MARCH 17

**Therapy Symposium ‐ SAM Salon AB**

***Management of Radiotherapy Patients with Implanted Cardiac Devices***

**MO‐A‐Salon AB‐01**

## Management of Radiotherapy Patients with Implanted Cardiac Devices

D Mihailidis,^1*^ J Prisciandaro^2*^

*Charleston Radiation Therapy Cons,^1^ Charleston, WV, University of Michigan,^2^ Ann Arbor, MI*

It has been twenty years since the AAPM published TG‐34 on cardiac pacemakers of older technology, which has been the standard document for clinical use, even today, for managing patients with pacemakers (ICPs). Management of radiotherapy patients with modern technology cardiac implantable electronic devices (CIEDs) has been widely published in literature without the provision of a new comprehensive and concise set of recommendations. This need is clearly evidenced by the numerous postings on the medical physics list server groups inquiring about advice on dealing with these devices during patient imaging and radiation treatments. As treatment delivery technologies (IMRT, SBRT, dose escalations, proton beams, etc.) and CIED technology advance, the need to address the management of patients with such devices receiving radiation treatment becomes increasingly important. As such, this session will provide updated guidance for caring for radiotherapy patients with CIEDs. Two presentations will be delivered during this session. The first will focus on the work of AAPM TG‐203 and provide a summary of the recommendations to the clinical user for management of patients with CIEDs when receiving RT. The second will focus on an institutional experience of managing patients with CIEDs.

**Learning Objectives:**

1. Review the purpose and function of CIEDs.
2. Provide a review on sources of potential malfunctions of modern CIEDs, including malfunction mechanisms from high‐LET radiation and transient effects attributed to medical imaging for radiotherapy.
3. Review the management of radiotherapy patients with cardiac devices.
4. Utilize recently available data and computation methods of out‐of‐field/peripheral dose by scattered photons and secondary neutrons estimate cumulative doses to CIEDs during treatment. Risk of failure associated with these doses will be discussed.
5. Provide comprehensive recommendations for management of radiotherapy patients with implanted cardiac devices from initial patient consultation to treatment delivery.

Dimitris Mihailidis (New Comprehensive and Practical Guidelines for Managing Radiotherapy Patients with Cardiac Devices —TG203)

Joann Prisciandaro (An Institutional Experience Managing the Care of Patients with CIEDs)

**Diagnostic Symposium ‐ SAM Salon CD**

***Release of Radioactive Patients / Patient Dose Monitoring Software***

**MO‐A‐Salon CD‐01**

## Release of Radioactive Patients / Patient Dose Monitoring Software

D Gress,^*^ D Hintenlang^2*^

*MD Anderson Cancer Center,^1^ Houston, TX, University Florida,^2^ Gainesville, FL*

The Nuclear Regulatory Commission provides several acceptable models for release of radioactive patients from radiation safety restrictions. The more computationally rigorous models can be used instead of traditional “rule of thumb” models to significantly reduce inconvenience to the patient and procedure cost. With hospital resources becoming scarcer and scrutiny toward radiation safety practices higher than ever, understanding and exploiting NUREG‐1556 radioactive patient release models can provide benefit to all stakeholders. Medical physicists are consulted for leadership in many issues associated with patient dose. This includes measurement, interpretation and, more recently, tracking of patient dose. The integration of the collected data with patients' medical records continues to evolve, with the medical physicist playing a central role in the success of these efforts. Commonly used metrics and their applicability for commonly used imaging techniques are discussed. The recording and disposition of these metrics continues to evolve. The recognized need to track patient dose has led both equipment vendors and independent software developers to introduce systems capable of recording and providing statistical analysis of patient doses delivered during imaging studies. A review of some commonly available patient dose‐tracking software systems is provided. Requirements and procedures for the review of, and incorporation of, patient dose into patient records is discussed with illustrative examples. This session highlights two critical and timely issues in radiation safety: release of radioactive patients from medical facilities and lifestyle restrictions, and patient dose tracking in X‐ray imaging. Fundamental physics and safety concepts are discussed and illustrated in clinical examples, along with more subtle, but important, considerations for the practicing medical physicist.

**Learning Objectives:**

1. Review risk‐based goals of appropriately timed release of radioactive patients from medical facilities and lifestyle restrictions.
2. Understand the reasonable patient release models provided by NRC guidance and their differences.
3. Introduce the various patient‐specific lifestyle considerations that can be used as a basis for accelerated release.
4. Review clinical case(s) demonstrating the impact of utilizing reasonable assumptions for accelerating radioactive patient release.
5. Understand regulatory requirements for recording and reporting patient dose associated with imaging studies.
6. Understand the applications and limitations of the various metrics used to quantify patient dose.
7. Develop an appreciation for the capabilities and limitations of commercially available patient dose tracking software packages.
8. Identify techniques and the steps required to include patient dose data in patients' medical records.

Dustin Gress (Release of Radioactive Patients from Restrictions Using NUREG‐1556) David Hintenlang (Patient Dose Tracking for Imaging Studies)

**Young Investigator Symposium Salon AB**

***Young Investigator Clinical Symposium***

**MO‐B‐Salon AB‐01**

## 2D Off‐Axis Corrections for EPID‐Based RapidArc QAs

F Jesseph,^*^ H Jin, S Ahmad

*University of Oklahoma Health Science Center, Oklahoma City, OK*

### Purpose

To investigate 2D off‐axis correction of EPID‐based RapidArc quality assurance (QA) at four photon energies by expanding on the research of Bailey et al.(1).

### Methods

A Varian TrueBeam STx with an aS‐1000 digital imaging panel was used to acquire RapidArc QA images for 17 patient plans totaling 27 arcs; each QA was performed at 6, 8, 10, and 15 MV. The Varian Portal Dose Image Prediction algorithm was used to create the comparison image for the EPID acquisition. The 2D correction map consists of a correction factor for each pixel, created by dividing the measured flood field readings by the predicted flood. The gamma passing rates of uncorrected EPID QAs were compared to those of 2D‐corrected QAs at 1%/1 mm, 2%/2 mm, and 3%/3 mm criteria. The two‐tailed Student's t‐test was employed to analyze the statistical significance between the uncorrected and 2D‐corrected measurements at the 95% confidence level.

### Results

The results showed a statistically significant improvement (p<0.05) under all criteria and energies when using the 2D correction matrix. The improvement ranges from 0.4% (15 MV) to 4.1% (8 MV) at 3%/3mm. When we focused exclusively on large field sizes (equivalent square≥15 cm), the improvement became more pronounced (1.3% (15 MV) to 6.7% (8 MV) at 3%/3 mm). In general, 8 and 10 MV showed better improvement than 6 and 15 MV

### Conclusion

A 2D correction matrix can substantially improve gamma passing rates for the RapidArc QAs. For 8 and 10 MV fields, the differences were more pronounced. The current Varian EPID panel calibration is insufficient; a 2D correction matrix would be recommended, especially when used for large fields. The jaw field size might be a superior predictor of the utility of a 2D correction.

1. Bailey et al. Med Phys. 2013;40:051704‐1.

**MO‐B‐Salon AB‐02**

## A Comprehensive and Efficient QA System for Validation of Photon Dose Calculation Accuracy

D Jacqmin,^*^ D McDonald, N Koch, J Peng, M Ashenafi, K Vanek

*Medical University of South Carolina, Charleston, SC*

### Purpose

A comprehensive and efficient QA system was created to validate the photon dose calculation accuracy of a treatment planning system (TPS). This automated system uses the series of tests suggested in AAPM TG‐53 and adds tests for validating MLC modeling.

### Methods

A 16 MV photon beam from a Varian 21eX linac was modeled using Philips Pinnacle^3^ v9.2. An automated QA system was created using MATLAB. The system generated a Pinnace script that automatically computed dose for a wide range of photon beams. This included symmetric fields, asymmetric fields, off‐axis fields, wedged fields, short and extended SSD fields, MLC‐shaped fields, and IMRT fields. The system also generated a Pinnacle3 script that produced planar dose maps at depths of 3, 7, 10, and 15 cm. The fields were delivered and absolute dose measurements were made with a MapCHECK2 diode array and Solid Water. The MATLAB system automatically compared the Pinnacle3 planar dose maps and measurements from MapCHECK2. Finally, the system produced a report with profile comparisons, gamma maps, and gamma passing rates (3%/3 mm) for each field and depth.

### Results

Gamma passing rates for each type of field were between 70% and 99%, with most fields having a passing rate greater than 95%. The poorest results were seen for small, asymmetric, offaxis fields. The tests also uncovered an issue with the MLC model, namely that the MLC leaf transmission parameter was overestimated. The parameter was adjusted accordingly, resulting in vastly improved MLC field QA and IMRT QA.

### Conclusion

This automated QA system allows an institution to perform the series of tests suggested in AAPM TG‐53 and quickly validate a TPS beam model during the commissioning process. The system provides valuable feedback to physicists by uncovering beam model errors and quantifying the limitations of the TPS dose calculation engine.

**MO‐B‐Salon AB‐03**

## Anthropomorphic Verification of the Eclipse AAA and Acuros Algorithms for Spine SBRT Treatments

M Grams,^*^ T Whitaker, L Fong de los Santos, D Brinkmann

*Mayo Clinic, Rochester, MN*

### Purpose

To test the ability of the Eclipse AAA and Acuros algorithms to accurately calculate dose in a highly heterogeneous medium typical of spine SBRT treatments.

### Methods

Two separate VMAT treatment plans using a 6 MV flattening filter‐free beam were delivered three times each to the cervical spine region of a Rando anthropomorphic phantom. Dosimetric measurements were made using GAFCHROMIC EBT3 film placed in a sagittal orientation at the midplane of the phantom and analyzed with FilmQA Pro software. Two separate PTVs were used, and the prescription dose was 24 Gy delivered in a single fraction with a maximum dose to the spinal cord of 10 Gy. The two PTVs were different, in that one partially encompassed the vertebral bodies and spinal cord (PTV 1) and the other completely encompassed them (PTV 2). Each plan was calculated using the Eclipse AAA and Acuros algorithms. Gamma analysis with a passing criterion of 3%/3 mm was used to assess the accuracy of plan delivery.

### Results

Gamma passing rates for PTV 1 using AAA were 98.1%, 97.9%, and 97.7%, and for Acuros were 98.2%, 97.6%, and 97.9%. Passing rates for PTV 2 were only 92.5%, 94.2%, and 91.3% using AAA, but improved to 95.9%, 96.6%, and 96.4% with Acuros. When delivered to a homogeneous Solid Water phantom, the plans had had gamma passing rates of 98.8% and 99.2% for PTV 1 and PTV 2, respectively.

### Conclusion

Treatment plans were delivered very accurately to the homogeneous phantom for either PTV; however, the lower passing rates associated with PTV 2 may be indicative of a problem with AAA in handling large heterogeneities in some plans. Improved agreement between calculation and measurement was seen with Acuros. Use of this algorithm, therefore, may be beneficial for spine SBRT and other highly heterogeneous targets, such as the nasopharynx.

**MO‐B‐Salon AB‐04**

## Are Output Measurements Always Necessary after CT Tube Replacement?

P Stauduhar,^*^ A Jones

*UT MD Anderson Cancer Center, Houston, TX*

### Purpose

TX regulations and the ACR require that CT radiation output be measured within 30 days of major service. The most common major service is tube replacement. We hypothesized that historical QC data could be used instead to determine if output measurements are necessary, reducing the need for costly output measurements.

### Methods

We reviewed 66 records of OEM tube replacements to determine with what frequency output falls outside specifications. We also conducted an experiment to verify that clinically significant output changes could be identified by comparing image noise in historical QC data with the same data after tube replacement. We used 30 days of historical QC data to establish a baseline noise level and 95% confidence interval (CI) for individual noise measurements. To simulate output changes, we acquired phantom images with our QC protocol while manually changing output (mA). We acquired ten images using the baseline output and ten images at each different “output”. We evaluated individual images and subsets of images at each “output” to determine if the system was within the manufacturer's specifications.

### Results

None of the 66 tube replacements resulted in an output change that exceeded specifications. Analysis of 30 days of historic QC data for our experimental system indicated a mean noise of 5.4 HU with 95% CI of 5.1–.5.7 HU. When using the mean noise of ten images acquired at each of the varying outputs, we were able to identify, with 100% sensitivity, images acquired at outputs outside manufacturer's specifications.

### Conclusion

The results of our review of historical OEM tube replacement data indicated the likelihood of output falling outside manufacturer's specifications is low. Considering this, it is likely that by using QC data from programs required by regulation and the ACR, physicists can reliably verify radiation output stability remotely instead of making physical measurements.

**MO‐B‐Salon AB‐05**

## Assessment of Target Localization Accuracy Estimated from Radiopaque Markers in Dynamic Tumor Tracking Irradiation

M Takamiya,^1*^ M Nakamura,^2^ M Akimoto,^2^ M Yamada,^2^ Y Matsuo,^2^ T Mizowaki,^2^ H Monzen,^2^ M Kokubo,^3^ M Hiraoka,^2^ A Itoh^1^

*Kyoto University Graduate School of Engineering,^1^ Kyoto, Kyoto, Kyoto University Graduate School of Medicine,^2^ Kyoto, Kyoto, Kobe City Medical Center General Hospital,^3^ Kobe, Hyogo*

### Purpose

To assess the target localization accuracy estimated from radiopaque markers in dynamic tumor tracking irradiation (DTT).

### Methods

This study included 15 patients who underwent four fractionated DTT for lung cancer. They had four or five radiopaque markers around a lung tumor. Before beam delivery, the orthogonal X‐ray fluoroscopic monitoring was done for 20–40 sec. First, 3D positions of the markers were detected from the X‐ray fluoroscopic images, and one marker was then assumed as a target. Next, the centroid of adjacent triple markers (PC), and the nearest (Pnear) and farthest (Pfar) marker positions from the target were determined from the remaining markers. Then, the distance between the target and each P at end‐exhalation on the initial day of the treatment was defined as the reference distance. The target position in every fraction was estimated by adding the reference distance to PC, Pnear, and Pfar. The |mean|+2 SD of difference between the actual and estimated target position during monitoring (E) were calculated for PC, Pnear, and Pfar. These procedures were repeated for the other markers. The overall (M), systematic (∑), and random (σ) errors (units; mm) were calculated with each method.

### Results

The median distances between the target and PC, Pnear and Pfar were 37, 28, and 65 mm, respectively. The value of M in the centroid, nearest, and farthest method was (1.6, 2.7, 2.5), (1.5, 2.3, 2.1), and (2.6, 4.5, 3.9) in the LR, SI, and AP directions, respectively. The value of ∑ was (1.4, 3.0, 2.3), (1.1, 2.1, 2.3), and (2.2, 4.6, 3.0) in the LR, SI, and AP directions, respectively. The value of σ was (0.8, 0.8, 1.1), (0.7, 0.8, 0.7), and (1.2, 1.2, 1.9) in the LR, SI, and AP directions, respectively.

### Conclusion

A single radiopaque marker near a target can be better surrogates of target position.

**MO‐B‐Salon AB‐06**

## Automated Treatment Planning System Dosimetric Quality Assurance

Taeho Kim,* Ryan Best, W. Tyler Watkins, Krishni Wijesooriya, Alan Aqualino, Bruce Libby, Jeffrey Siebers

*University of Virginia Health Systems, Charlottesville, VA*

### Purpose

To describe an automated dosimetric quality assurance (QA) procedure for the Pinnacle^3^ radiotherapy treatment planning system (TPS) for use in commissioning validation and annual TPS quality assurance.

### Methods

A Pinnacle QA script was created which functions in the clinical treatment planning mode, as opposed to the commissioning mode commonly used for data comparison. The script iterates over the different treatment machines, modalities, energies, treatment devices, jaw, and MLC defined field sizes, and SSDs which correspond to annual water phantom measurements and/or prior commissioning results. Sequentially, the script created the beams, computed dose, exported the computed dose profiles, and compared them to measured dose data, which is normalized to report dose/MU. Automated analysis included graphical profiles and dose differences, as well as distance‐to‐agreement values, uniformities, penumbrae, depth‐dose metrices, and other associated dosimetric metrices. A single verification report for physicist review and evaluation was generated. The script was run for dose matrix resolutions corresponding to those used for clinical planning (2–4 mm). An additional automated suite of geometric tests and typical patient plans is run in conjunction with these dosimetric tests to complete the TPS QA.

### Results

The automated dosimetric QA procedure enabled rapid one‐click comparison of measured dose and planned dose. The verification report provided physicists a concise and organized summary of the planned dose accuracy. Results were generally within TG‐53/ and ESTRO Booklet #7 criteria (±2%,±2 mm); however, dose deviations at depths≤Dmax varied and exceeded 5% for some dose‐voxel resolutions and absolute voxel placements.

### Conclusion

An automated dosimetric QA procedure for comparison of measured and planned dose was developed and implemented for the clinical acceptance of the Pinnacle^3^ treatment planning system. This proposed procedure will be a valuable tool for routine clinical dosimetric TPS QA required during TPS upgrades and annual QA.

**MO‐B‐Salon AB‐07**

## Developing a Novel Method to Analyze GAFCHROMIC EBT2 Films in Intensity‐Modulated Radiation Therapy Quality Assurance

Y Hu,^1*^ Y Wang,^1^ G Fogarty,^2^ G Liu^2^

*Genesis Cancer Care,^1^ Darlinghurst, NSW, Genesis Cancer Care,^2^ Crows Nest, NSW, Australia*

### Purpose

Recently individual IMRT QA have been more and more performed with GAFCHROMIC EBT series films processed in Red‐Green‐Blue channel. However, the efficiency of this method is relatively low, as for each new box of film a calibration curve must be established. In this study, the authors proposed a novel method to process the GAFCHROMIC EBT series: to use the 16‐bit gray‐scale channel to process the film, which greatly increases the efficiency and accuracy of the QA procedure.

### Methods

The authors firstly calibrated the GAFCHROMIC EBT2 film using gray‐scale channel in three dose ranges, and established a Pixel value‐to‐dose calibration curve. This calibration curve was implemented into an in‐house film analyzing software. This method of film processing was then used to perform the QA of 743 IMRT cases across two radiation therapy centers, and the QA results were compared with ionization chamber measurements.

### Results

The authors find that within a dose range of 0 to 600 cGy, the GAFCHROMIC EBT2 film presents a perfect linear PV‐to‐dose calibration curve when processed in the gray‐scale channel. This fact greatly improves the accuracy and efficiency of calibrating GAFCHROMIC EBT film, as with a known linear relationship, only two data points are needed to establish the calibration curve; the fitting error is also significantly reduced. The whole procedure can be further simplified if the film is used for relative measurements only. Among the 743 clinical cases tested, 740 cases passed the 3%3 mm gamma‐function assessment and the results agreed well with ion chamber measurements. The failed three were found to originate from human mistakes.

### Conclusion

The authors confirm that the novel method is effective and efficient. Clinical testing has shown consistent results, proving that this method can be used to replace the conventional R‐G‐B method to analyze GAFCHROMIC EBT2 films in IMRT QA.

**MO‐B‐Salon AB‐08**

## Generation and Applications of 3D‐Printed, Patient‐Specific Devices for Proton Therapy

X Ding,^1*^ A Witztum,^1^ M Reiche,^2^ B Teo,^1^ L Yin,^1^ H Lin,^1^ A Olszanski,^1^ J Fiene,^2^ A Kassaee^1^

*Perelman Center for Advanced Medicine,^1^ University of Pennsylvania, Philadelphia, PA, Department of Mechanical Engineering and Applied Mechanics,^2^ University of Pennsylvania, Philadelphia, PA*

### Purpose

We introduce a simple method to use 3D printers to manufacture patient‐specific bolus and supporting devices created in a treatment planning system (TPS) for proton therapy.

### Methods

One brain and one left partial breast proton patient were included in this study. During the planning process, boluses were designed in order to optimize the dose distribution. For the brain patient, a 5 cm thick bolus was created to shift the proton range in order to treat the superficial nasal area using pencil beam scanning (PBS) technique. For the left partial breast patient, a custom bolus, which can be attached to the breast board, was generated. DICOM RT structure set files of bolus structures were exported from the TPS and converted to stereolithography format (STL) for 3D printer. The relative stopping power of the 3D printed material, ABSplus‐P430, were measured using the Zebra multilayer ionization chamber system.

### Results

The geometric difference between TPS bolus structure and corresponding STL structure was within 0.2 mm based on analysis performed using MATLAB. The relative stopping power of the material, ABSplus‐P430, was 1.05 which is almost water‐equivalent. From planning perspective, for the partial breast patient, with the option of using PBS plan with 3D bolus, the skin maximum dose and nipple dose were significantly reduced. Homogeneity of the PBS proton plan was also greatly improved compared to the passive scattering plan. It not only gives us an option to treat using PBS technique, but also can be used as a supporting and immobilization device.

### Conclusion

3D printers can be used to accurately manufacture patient‐specific boluses from the TPS. The ability to create and construct complex patient‐specific bolus structures provides a new dimension in creating optimized quality treatment plans.

**MO‐B‐Salon AB‐09**

## MammoSite Patient Quality Assurance Catheter Check

J McGlade,^1*^ C Goldberg^2^

*University of Pennsylvania,^1^ Philadelphia, PA, Main Line Health,^2^ Bryn Mawr, PA*

### Purpose

To quantify the dose changes to planned target volumes (PTVs) and organs at risk (OARs) due to catheter rotation or misidentification using MammoSite Multi‐Lumen Balloon (MLB) Brachytherapy.

### Methods

Data from 27 previously treated patients were analyzed using Oncentra treatment planning. Treatment plans were based on CT scans using a GE LightSpeed with 1.25 mm slice thicknesses. For each patient, two new treatment plans were generated, each with a catheter rotation and otherwise identical treatment parameters. The dwell‐time optimizations from the original plan were used to simulate the catheter rotations. The dose‐volume histogram was analyzed for each plan and compared with the sample population to determine the mean distributions and standard deviation.

### Results

Coverage of the PTV was maintained for any rotation of the MLB plan, with a mean change of 2.2%. The mean changes to the skin and chest wall were 9.5% and 16.6%, respectively. While the mean dose changes to these critical organs do not appear extreme, instances of overdosing of as much as 58.3% and 79.9% were observed. The mean change to the V150 and V200 were 1.13 and 0.81 cubic centimeters, respectively.

### Conclusion

During simulation, marker wires are placed in each channel by the physicist or a member of the Radiation Oncology team. There is a potential for the marker wires to be placed in the incorrect channel, which will cause the treatment to be delivered as if the balloon is rotated. The balloon may also rotate due to movement in the patient. These factors can both lead to the results above. For patients with minimum OAR separation distances of less than 9 mm or treatment plans with several dwell locations turned off, we recommend a CT scan before the first fraction of MammoSite treatment as a check against an erroneous catheter rotation or misidentification.

**MO‐B‐Salon AB‐010**

## Site‐Specific Tolerance Tables with a Customized Indexing Device to Improve Patient Position Repeatability for Radiotherapy Treatments

K McCullough,^1*^ J James,^1^ M McCullough,^2^ B Wang^1^

*University of Louisville,^1^ Louisville, KY, [notprovided],^2^ Louisville, KY*

### Purpose

To design a set of site‐specific treatment tolerance tables with a customized indexing system that will insure accurate patient setup with minimal workflow interruption.

### Methods

A retrospective analysis was performed on a total of 74 patients and 1428 treatments, separating them into nine distinct categories based on site of treatment and method of immobilization. Couch parameter tolerance tables were designed to encompass 95% of treatments and were generated by calculating the standard deviation of couch vertical, longitudinal, and latitudinal values using the first day of treatment as a baseline. A custom device was then constructed to be used for indexing patients who lacked a means of indexing, and the system was implemented on a linear accelerator at our cancer center in order to determine its feasibility.

### Results

During this trial period, we collected data from 188 fractions from 13 patients and five sites, and analyzed the number of treatments that would have been out of tolerance and whether the tolerances or setup techniques should be adjusted. Of those 188 fractions, 52 (28%) would have required physics intervention if the original baseline was kept for further reference and 29 (15%) if the baseline was changed after every tolerance violation. The use of the indexing device allowed for pelvis sites to be set up with results similar to that of wing board/immobilized patients from the initial study (e.g., 2.02 cm vs. 1.64 cm average deviation in longitude). The results also indicate an increase in tolerance size for some sites may be necessary if issues in setup cannot be addressed.

### Conclusion

Further data collection and refinements to patient indexing technique are required before the tolerance tables are implemented. Our initial results show promise that a system can be developed that will enhance patient safety without hindering clinical efficiency.

**MO‐B‐Salon AB‐11**

## Validation of the Monte Carlo Algorithm for Electrons in Treatment Planning

S Enright,^1*^ A Asprinio,^2^ L Lu^2^

*University of Miami,^1^ Coral Gables, FL, South Florida Radiation Oncology,^2^ Stuart, FL*

### Purpose

This study was conducted to validate the Monte Carlo algorithm for electron therapy in the Eclipse treatment planning system. Irradiation with electrons offers the advantages of dose uniformity in the target volume and in minimizing the dose to deeper healthy tissue. Using the Monte Carlo algorithm will improve dose accuracy in regions with heterogeneities.

### Methods

Dose distributions from film measurements were compared to dose distributions from Electron Monte Carlo, and Pencil Beam algorithms in Eclipse. These measurements were obtained for 5 MeV, 9 MeV, and 12 MeV electrons. All phantoms studied were imported into Eclipse by CT scan. A 1 cm thick solid water template with one square and two circular holes for bonelike and lung‐like plugs was used. Each configuration had either all bone or all lung plugs. Film measurements were taken at two depths for each energy. The depths used were the depth of maximum dose (dmax), and a depth greater than dmax. Ionization chamber measurements were also obtained at different locations and were used for comparison and to verify the film measurements.

### Results

The accuracy of Electron Monte Carlo was compared to that of Pencil Beam. The dose from Monte Carlo agreed better than that from Pencil Beam for all configurations and depths. The pass rate for Monte Carlo was in the 80%−99% range, where the pass rate for Pencil Beam was as low as 10.76%. Ionization chamber measurements verified that the dose given from the film was accurate.

### Conclusion

The dose distribution from Monte Carlo agreed much better with the measured dose from the film when compared to the Pencil Beam algorithm. The pass rates for Monte Carlo were significantly higher than those from Pencil Beam. Monte Carlo should be used over Pencil Beam for regions with heterogeneities.

**Professional Symposium Salon AB**

***ABR Exam Update, Job Interview Improvement ‐ A Fun, Interactive Session for Interviewers and Interviewees***

**MO‐C‐Salon AB‐01**

## ABR Exam Update, Job Interview Improvement — A Fun, Interactive Session for Interviewers and Interviewees

J Seibert,^1*^ R Miller,^2*^ M Meineke^3*^

*UC Davis Medical Center,^1^ Sacramento, CA, Northwest Medical Physics Center,^2^ Seattle, WA, Hardin Memorial Hospital,^3^ Elizabethtown, KY*

J. Anthony Seibert ‐ **ABR Examination Update Initial Certification and Maintenance of Certification of Medical Physicists**

The American Board of Radiology provides opportunities for individuals trained in the field of clinical medical physics to acquire and maintain certification as a Diplomate of the ABR in the fields of Therapeutic Medical Physics, Diagnostic Medical Physics, and Nuclear Medical Physics. Eligibility requirements for Initial Certification (IC) examinations are described, as well as the exam process, including the Part 1 (General Medical Physics and Clinical Exam), Part 2 (specific qualification exam for a designated field of Medical Physics), and Part 3 (Oral Exam for those who pass Part 2). Upon receiving initial certification, each Diplomate is automatically enrolled in the Maintenance of Certification (MOC) program, which builds on the validity of the IC process with six essential competencies of clinical practice (initially developed in residency training) through a four‐part evaluation process. The MOC program and processes are described with respect to continuous look‐backs and time periods necessary to successfully maintain certification.

**Learning Objectives:**

1. Understand the processes necessary to achieve ABR certification in Medical Physics.
2. Learn about recent changes to the ABR exam process.
3. Illustrate the need for MOC and describe the evaluation competencies.

Robin Miller (Job Interview Improvement — A Fun, Interactive Session for Interviewers and Interviewees)

Matthew Meineke (Job Interview Improvement — A Fun, Interactive Session for Interviewers and Interviewees)

This session will address professional aspects of the certification process and interviewing for clinical medical physics positions. It will include the latest updates relevant to medical physics certification through the American Board of Radiology processes, including initial certification, maintenance of certification, and the examination process. The second portion of the session provides a relaxed and fun session for those seeking to improve their interviewing skills. You got your foot in the door, they liked your resume, and thought you looked interesting enough to bring out to meet face to face. How do you showcase your skills, while simultaneously showing them how well you would mesh with their current team? How do you respond to these newly popular questions that seem more interested in your feelings than in your technical skills? How do you pick up on the details that might clue you in that this potential employer isn't right for you? Our session will give you concrete advice with examples to give you the tools you need to be successful in your next interview.

**Learning Objectives:**

1. How to highlight your skill set for potential employers.
2. How to respond to behavior based (vs. technical based) interview questions.
3. How to evaluate potential employers as a good fit for you.

J. Anthony Seibert (ABR Examination Update: Initial Certification and Maintenance of Certification of Medical Physicists)

Robin Miller (Job Interview Improvement — A Fun, Interactive Session for Interviewers and Interviewees)

Matthew Meineke (Job Interview Improvement — A Fun, Interactive Session for Interviewers and Interviewees)

**Professional Symposium ‐ SAM Salon CD**

***Leadership and Project Management***

**MO‐C‐Salon CD‐01**

## Safety Culture Leadership: What Can We Learn from High Reliability Organizations? / How I Learned to Love Databases and Stop Abusing Excel

D Jordan

*University Hospitals Case Medical Center, Shaker Heights, OH*

**Safety Culture Leadership: What Can We Learn from High Reliability Organizations?**

In 2011, the U.S. Nuclear Regulatory Commission enacted its Safety Culture Policy Statement requiring licensees to establish and maintain a “positive safety culture.” Since that time, the NRC and state radiation regulatory agencies have begun to clarify how they will implement this policy in rule‐making, inspection, and so forth. There is a great deal of room for interpretation about what it means to have a safety culture and how to achieve it. Medical physicists, in their common roles as clinical physicists and/or Radiation Safety Officers, are responsible for providing leadership in these programs, including the creation of policies and procedures and monitoring compliance within medical organizations. To provide the clinical physicist and Radiation Safety Officer with specific and actionable ways to create and maintain safety culture, this talk will examine the characteristics of High Reliability Organizations (HRO), such as naval aviation and nuclear power, that are inherently very dangerous but have established extraordinary track records of safety. These characteristics result from practices that are adopted by the leaders and taught and reinforced to all members of the organization. The behaviors that make an organization an HRO inherently create and sustain strong positive safety cultures.

**”How I Learned to Love Databases and Stop Abusing Excel”**

Spreadsheets are a common approach to storing lists and data that would be more useful and functional in a database. The problem is that it is very easy to create a blank spreadsheet to enter and manipulate basic data. Many spreadsheets become large and unwieldy and contain huge amounts of mission‐critical information before their users begin to encounter limits on what the spreadsheet format can handle. Relational databases, on the other hand, are powerful tools, but they usually require specialized knowledge to create and maintain. This overhead seems daunting and unnecessary for a “lightweight” task, and so spreadsheets proliferate. This lecture will explore the concepts of database design and implementation, and review how to recognize an application where a database is a superior choice to spreadsheets for data management. Participants should gain the ability to define the requirements of a database development project, discuss requirements and features with database developers and software vendors, and, if motivated, to begin learning the functional aspects and syntax of a specific database environment.

**Therapy Symposium ‐ SAM Salon AB**

***Forthcoming Task Group Reports***

**MO‐D‐Salon AB‐01**

## Forthcoming Task Group Reports

A Olch

*Children's Hospital of LA, Los Angeles, CA*

The dosimetric impact from devices external to the patient is a complex combination of increased skin dose, reduced tumor dose, and altered dose distribution. Although small monitor unit or dose corrections are routinely made for blocking trays, ion chamber correction factors, or tissue inhomogeneities, the dose perturbation of the treatment couch top or immobilization devices are often overlooked. These devices also increase surface dose, an effect which is also often ignored or underestimated. These concerns have grown recently due to the increased use of monolithic carbon fiber couch tops, which are optimal for imaging for patient position verification but cause attenuation and increased surface dose compared to the “tennis racket” style couch top they often replace. Also, arc delivery techniques have replaced stationary gantry techniques, which cause a greater fraction of the dose to be delivered from posterior angles. A host of immobilization devices are available and used to increase patient positioning reproducibility, and these also have attenuation and skin dose implications, which are often ignored. This report of Task Group 176 serves to present a survey of published data that illustrates the magnitude of the dosimetric effects of a wide range of devices external to the patient. The report also provides methods for modeling couch tops in treatment planning systems so the physicist can accurately compute the dosimetric effects for indexed patient treatments. Both photon and proton beams are considered. A discussion on avoidance of high‐density structures during beam planning is also provided. An important aspect of this report are the recommendations we make to clinical physicists, treatment planning system vendors, and device vendors on how to make measurements of skin dose and attenuation, how to report these values and, for the vendors, an appeal is made to work together to provide accurate couch top models in planning systems.

**Learning Objectives:**

1. What are the dosimetric effects of couch tops?
2. What are the dosimetric effects of immobilization devices?
3. How can one model couch tops in the treatment planning system?
4. How can one measure attenuation and surface dose changes due to external devices?

**MO‐D‐Salon AB‐02**

## Forthcoming Task Group Reports

R Siochi

*University Of Iowa, Iowa City, IA*

The transfer of data along the chain from imaging to treatment in radiotherapy has been a source of errors. TG201 has written a rapid communication to deal with this issue, and is also in the process of writing a full report that details the typical environments and data communication models in radiotherapy, along with recommendations on the QA tests, philosophies, and methodologies. Single database systems from single vendors, as well as systems comprised of a network of mixed vendors, will be explored in terms of the exchange of data among various subsystems. Methods for analyzing one's data transfers (e.g. the data transfer matrix), the associated risks (e.g. prospective fault tree analysis), and the required tests, will be presented. Along with these tests, the design of more robust clinical workflows involving data transfer procedures will be explained.

**Learning Objectives:**

1. Understand the various data communication models and identify the one used in your clinic.
2. Learn strategies for analyzing data transfer risks.
3. Understand the principles of robust clinical workflow design as it relates to data transfer procedures.

**Diagnostic Symposium ‐ SAM Salon CD**

***Refresher and What's New in PET/Gamma Camera Cardiac Imaging***

**MO‐D‐Salon CD‐01**

## Refresher and What's New in PET/Gamma Camera Cardiac Imaging

O Mawlawi,^1*^ R Quaife^2*^

*MD Anderson Cancer Ctr.,^1^ Houston, TX, University of Colorado,^2^ Aurora, CO*

This talk will cover basic PET image acquisition and formation. Description of data formats, corrections, and quality control will be provided. The talk will also cover recent developments in PET imaging such as TOF, resolution recovery, continuous Bed motion, reconstruction, and PET/MR. Imaging of cardiovascular disease is rapidly progressing to a disease‐specific quantitative diagnostic methodology. This movement is highly dependent upon physics‐based improvements in reconstruction algorithms, and acquisition hardware and software, which are all focused on the goals of greater accuracy and reduced radiation dose for our patients. This push for quantitative cardiac assessment includes development of disease‐specific radiotracers. These advances in tracers and technology have stimulated development of merged imaging modalities aimed at rapid imaging of both structure and function of the heart. This session includes a review of fundamental concepts in emission imaging. Recent developments in both applied physics and the clinical realm are also featured.

**Learning Objectives:**

1. Understand the PET imaging chain.
2. Become familiar with PET QA/QC.
3. Learn the new developments in the field of PET imaging.
4. Understand current attenuation correction methods for SPECT/CT and PET/CT in cardiac imaging.
5. Review image quality assurance methods necessary for accurate diagnoses of myocardial perfusion and perfusion reserve.
6. Review new receptor based radionuclide tracer for cardiac pathophysiology.
7. Understand multimodality imaging of cardiovascular disease.

Osama Mawlawi (Refresher and What's New in PET)

Robert Quaife (Emission Imaging in Cardiology)

**TUESDAY, MARCH 18**

**Therapy Symposium ‐ SAM Salon AB**

***The Role of Independent Audits in Quality***

**TU‐A‐Salon AB‐01**

## The Role of Independent Audits in Quality

A Beavis,^1*^ D Letourneau,^2*^ S Kry^3*^

*Queens Centre for Oncology & Haematology,^1^ Cottingham, Princess Margaret Hospital,^2^ Toronto, ON, MD Anderson Cancer Ctr.,^3^ Houston, TX*

A number of guidance documents are aimed at supporting quality care in radiation therapy. However, much can be learned by evaluating and comparing practice patterns, determining areas of deficiencies, and providing support to programs. In the United Kingdom, an audit program was established as part of a national effort to standardize and improve the quality of care. The program includes site visits by physicists from other centers where measurements are performed along with a review of the program. These visits have led to a variety of improvements nationally. In Ontario, the Cancer Care Ontario program has a similar goal of improving quality throughout the province. The collaborative quality assurance (CQA) program was established to assess the planning and delivery quality of both static‐gantry IMRT and arc‐based IMRT delivery (e.g., VMAT and TomoTherapy) through a standardized end‐to‐end test which includes planning exercise and a site visit. 14 centers have participated in the program in the first two years, with tests completed for both prostate and head and neck cancer. In addition to results comparing the planned to measured dose distributions, feedback is provided on the planning practice, phantom set up, plan delivery, beam model and machine performance. Extension of the CQA funding to 2017 enabled a program designed to assess change in planning and delivery performance through repeat visits. In North America, the quality of irradiation and beam commissioning is evaluated for radiotherapy centers participating in NIH‐sponsored clinical trials. These remote dosimetry and phantom programs have supported assuring quality in trials with 1809 and 236 participants throughout North America and elsewhere in the world, respectively. These North American audits have not only improved the accuracy of dose delivery for clinical trial participants, but have also yielded secondary benefits to Radiation Oncology practice and patient safety.

**Learning Objectives:**

1. Learn about a mechanism to improve the quality of care through an audit program in the United Kingdom.
2. Understand the value of comparing standard measurements for an advanced technology directly with other programs as done in Ontario, Canada.
3. Learn about the mechanisms available in the US to evaluate quality compared to other centers in support of quality in clinical trials and their impact beyond auditing.

Acknowledgements: NIH CA10953‐45 (RPC), CA081647 (ATC) and Cancer Care Ontario

Andrew Beavis (Quality in the UK: Evolution of an Audit Program)

Daniel Letourneau (Provincial Collaborative IMRT QA program: The Cancer Care Ontario Experience)

Stephen Kry (Beyond auditing: What we have learned from phantom credentialing for clinical trials)

**Diagnostic Symposium ‐ SAM Salon CD**

***Ultrasound ACR Accreditation: Roles of the Medical Physicist***

**TU‐A‐Salon CD‐01**

## Ultrasound ACR Accreditation: Roles of the Medical Physicist

S Larson,^1*^ N Hangiandreou^2*^

*University Michigan Medical Center,^1^ Ann Arbor, MI, Mayo Clinic,^2^ Rochester, MN*

Recent revisions to the American College of Radiology's ultrasound accreditation program requirements (http://www.acr.org/~/media/ACR/Documents/Accreditation/US/Requirements.pdf) may alter the way many ultrasound practices view quality control (QC) in ultrasound. Unlike the previous ultrasound QC recommendations, the new requirements consist of multiple components which define a comprehensive QC program. The main components of the QC program are acceptance testing, the annual survey, semiannual (or preferably quarterly) routine QC tests, and preventive maintenance. A comparison to ACR QC requirements in other modalities suggests that QC should be a team effort, with the QC program design and oversight, acceptance testing and annual survey being the responsibility of the medical physicist. The routine QC would be the responsibility of an imaging technologist, and preventive maintenance would be the responsibility of a service engineer. The best approach to meeting the new ACR ultrasound requirements is a team approach, with all of these professionals working in concert to create and sustain an effective QC program. The ACR strongly recommends that the QC program be supervised by a qualified medical physicist. As a result, we expect physicists to be asked to play a major role in the design and supervision of these QC programs. The new ACR accreditation program requirements provide no specifics regarding acceptance testing, except that that it should include the annual tests in order to provide baseline data. For the annual survey, seven tasks are specifically required: physical/mechanical inspection, uniformity/artifact survey, geometric accuracy, system sensitivity, scanner display performance, primary interpretation display performance, and evaluation of the QC program. Two additional annual tasks are optional: contrast resolution and spatial resolution. The routine QC tasks include: physical/mechanical inspection, uniformity/artifact evaluation, scanner display performance, primary interpretation display performance, and geometric accuracy (the last is only for mechanically scanned transducers). The specifics of how to perform these tests and analyze the results are not provided, and are left in the hands of the person designing the QC program. The purpose of this session is to provide medical physicists with the tools they need to meet the new ACR requirements for ultrasound accreditation. The first part of this session will cover how to perform an annual survey of ultrasound equipment. The second part will address the physicist's role in implementing and supervising a continuous ultrasound QC program.

**Learning Objectives:**

1. Understand the new ACR requirements for ultrasound quality control and the roles of the medical physicist.
2. Learn the basics of how to perform an annual survey of an ultrasound system.
3. Understand the physicist's role in implementing and supervising the routine QC testing process.

Sandra Larson (ACR Requirements and the Annual Survey)

Nicholas Hangiandreou (ACR Requirements for Routine Quality Control)

**Professional Symposium Salon AB**

***Regulatory Update***

**TU‐B‐Salon AB‐01**

## Regulatory Update

G White,^1*^ L Fairobent^2*^

*Colorado Associates in Medical Phys,^1^ Colorado Springs, CO, AAPM,^2^ College Park, MD*

**Medical Reimbursement Economics 201: Foundations, This Year's Changes and the Hazy Crystal Ball Look Forward** ‐ Gerald A. White, M.S.

The year 2014 will bring a number of changes in the reimbursement environment for both Radiation Oncology and Imaging services. This talk will review the underlying structure of the Medicare‐based systems for both hospital and free‐standing facilities and the influence of the American Medical Association on the determination of relative values for services. Specific changes for CPT codes in 2014 will be presented and explained, with guidance as to proper use. The 2014 reimbursement levels for new and revised codes will be compared to previous valuations. Lastly, future changes in CPT codes and reimbursement levels will be discussed. There will be ample time for questions and discussions on the application details of the CPT codes related to Medical Physics.

**Be careful what you say, big brother is listening** ‐ Lynne Fairobent

NRC is moving forward on publication of changes to 10 CFR Part 35. The proposed rule addresses three ongoing rulemaking projects and several other related topics. First, this rule proposes amendments to the reporting and notification requirements for a medical event (ME) for permanent implant brachytherapy. Second, the rule proposes changes: (1) to the training and experience (T&E) requirements for authorized users (AUs), medical physicists, Radiation Safety Officers (RSOs), and nuclear pharmacists; (2) to the requirements for measuring molybdenum contamination and reporting of failed technetium and rubidium generators; and (3) to allow Associate Radiation Safety Officers (ARSOs) to be named on a medical license. Third, the rule proposes changes to address a request filed in a petition for rulemaking (PRM) (PRM‐35‐20) to exempt certain board‐certified individuals from certain T&E requirements (i.e., “grandfather” these individuals) so that they may be identified on a license or permit for materials and uses that they performed on or before October 24, 2005, the expiration date of the former Subpart J of Part 35 which contained the prior T&E requirements. After discussing the proposed changes, there will be ample time for questions and discussions on the impact of these changes on the practice of medicine.

**Learning Objectives:**

1. Participants will become familiar with key regulation and guidance changes for the use of radioactive materials in medicine that are under consideration.
2. Participants will provide feedback on proposed changes to presenters.
3. Participants will be prepared to facilitate engagement of their facility and colleagues in the ongoing dialogue about changes.

Gerald White (Medical Reimbursement Economics 201: Foundations, This Year's Changes and the Hazy Crystal Ball Look Forward)

Lynne Fairobent (Be careful what you say, big brother is listening)

